# Matrix stiffness regulates macrophage polarisation via the Piezo1‐YAP signalling axis

**DOI:** 10.1111/cpr.13640

**Published:** 2024-03-31

**Authors:** Feng Mei, Yaru Guo, Yu Wang, Yingying Zhou, Boon Chin Heng, Mengru Xie, Xiaofei Huang, Shihan Zhang, Shuai Ding, Fangyong Liu, Xuliang Deng, Lili Chen, Cheng Yang

**Affiliations:** ^1^ Department of Stomatology, Union Hospital, Tongji Medical College Huazhong University of Science and Technology Wuhan China; ^2^ School of Stomatology, Tongji Medical College Huazhong University of Science and Technology Wuhan China; ^3^ Hubei Province Key Laboratory of Oral and Maxillofacial Development and Regeneration Wuhan China; ^4^ Department of Geriatric Dentistry Peking University School and Hospital of Stomatology Beijing China; ^5^ Department of Orthodontics Peking University School and Hospital of Stomatology, National Clinical Research Center for Oral Diseases & National Engineering Laboratory for Digital and Material Technology of Stomatology & Beijing Key Laboratory of Digital Stomatology Beijing China; ^6^ NMPA Key Laboratory for Dental Materials, Department of Dental Materials & Dental Medical Devices Testing Center Peking University School and Hospital of Stomatology Beijing China; ^7^ Central Laboratory Peking University School and Hospital of Stomatology Beijing China

## Abstract

Macrophages play a pivotal role in the immunological cascade activated in response to biomedical implants, which predetermine acceptance or rejection of implants by the host via pro‐ and anti‐inflammatory polarisation states. The role of chemical signals in macrophage polarisation is well‐established, but how physical cues regulate macrophage function that may play a fundamental role in implant‐bone interface, remains poorly understood. Here we find that bone marrow‐derived macrophages (BMDM) cultured on polyacrylamide gels of varying stiffness exhibit different polarisation states. BMDM are ‘primed’ to a pro‐inflammatory M1 phenotype on stiff substrates, while to an anti‐inflammatory M2 phenotype on soft and medium stiffness substrates. It is further observed that matrix stiffening increases Piezo1 expression, as well as leads to subsequent activation of the mechanotransduction signalling effector YAP, thus favouring M1 polarisation whilst suppressing M2 polarisation. Moreover, upon treatment with YAP inhibitor, we successfully induce macrophage re‐polarisation to the M2 state within the implant site microenvironment, which in turn promotes implant osseointegration. Collectively, our present study thus characterises the critical role of the Piezo1‐YAP signalling axis in macrophage mechanosensing and stiffness‐mediated macrophage polarisation and provides cues for the design of immuno‐modulatory biomaterials that can regulate the macrophage phenotype.

## Graphical abstract

Matrix stiffening promotes M1 macrophage polarisation via the Piezo1‐YAP signalling axis. The application of YAP inhibitor successfully reverses the polarisation of macrophages towards anti‐inflammatory M2 phenotype and improves implant osseointegration.

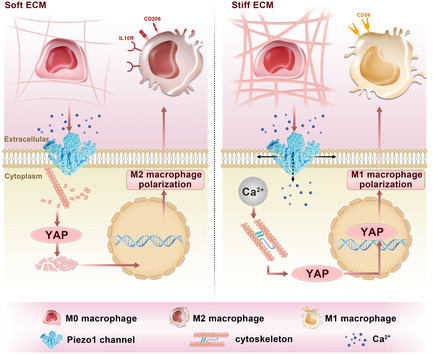

## 1 INTRODUCTION

Titanium (Ti) and Ti‐alloy implants have been widely applied in orthopaedics and dentistry since the early 1970s.^1^, ^2^ In general, Ti is considered to be a highly biocompatible metallic material for implantation and surface coatings have been applied to further improve its biocompatibility.^3^ However, Ti implants are not completely devoid of the capacity to provoke inflammatory responses. For example, wear particles and degradation by‐products from implants may elicit a local chronic inflammatory response and peri‐prosthetic osteolysis, eventually leading to inflammation‐related implant failures.^4^, ^5^ After implantation, a sequence of immune events dominated by macrophages is triggered and the macrophage phenotype predetermines the fate (function vs. failure) of implants. Therefore, modulation of macrophage phenotype towards an anti‐inflammatory (M2)‐like state has been considered a therapeutic approach for mitigating implant failures.^6^, ^7^ Work from the past decade has tremendously expanded our knowledge on the soluble, diffusible signals involved in macrophage polarisation.^8^ However, considering the mechanical properties of Ti implants and its surrounding hard tissues after implantation, physical cues, such as environmental stiffness, might play a more important role in controlling macrophage phenotype and deep research on the underlying molecular mechanisms could provide new therapeutic targets.

Matrix stiffness has been recognised as an important regulator of cell function, which can influence mesenchymal stem cell differentiation,^9^ endothelial tip cell specialisation,^10^ neutrophil morphology and migration.^11^ In addition, there have also been studies on the effects of stiffness on macrophage phagocytosis and elasticity, but the underlying mechanisms are unclear.^12^ Piezo1, a novel class of mechanosensory calcium channels, is critical for sensing and transducing various mechanical stimuli.^13^, ^14^ It has been found to play a key role in inflammation by acting as a mechanosensor of pressure and shear stress in myeloid cells that are recruited to the heart, lung and tumours, which further confirmed its role as a key mediator of inflammation in response to mechanical stimuli.^15^, ^16^ Yes‐associated protein (YAP) is the predominant effector molecule of the mammalian Hippo pathway and regulates monocyte adhesion to inflamed endothelium, as well as macrophage activation.^17^, ^18^ A recent study demonstrated that Piezo1 could modulate endothelial atherogenic inflammatory responses via regulation of YAP/TAZ activation.^19^ Moreover, Piezo1‐YAP signal transduction is involved in the epithelial‐mesenchymal transition of mucosal epithelial cells,^20^ osteogenic differentiation of aortic valve interstitial cells,^21^ and metastasis of ovarian cancer cells.^22^ Hence, we hypothesise that the Piezo1‐YAP signalling axis may also play a crucial role in macrophage function. However, there have been no relevant studies to date.

In this study, we have explored the effects of matrix stiffness on macrophage polarisation and further investigated the underlying molecular mechanisms involved. Bone marrow‐derived macrophages (BMDM) were ‘primed’ to a pro‐inflammatory M1 phenotype on stiff substrates, while soft and medium stiffness substrates favoured an anti‐inflammatory M2 phenotype. Mechanistically, matrix stiffening upregulated Piezo1 expression, as well as YAP expression and its nuclear accumulation, thus favouring M1 polarisation whilst suppressing M2 polarisation. Eventually, macrophage polarisation to the M1 phenotype on a stiff substrate was reversed by YAP deficiency and improved implant osseointegration was subsequently achieved through YAP inhibition. Together, this study thus demonstrated that matrix stiffening regulated macrophage polarisation via the Piezo1‐YAP signalling axis. Moreover, we have also identified new molecular targets that promote biomaterial‐bone integration by reversing macrophage polarisation towards M2 phenotype within a stiff environment, and provided cues for immuno‐modulatory biomaterial design in the field of tissue engineering and regenerative medicine.

## 2 RESULTS

### 2.1 Macrophages polarise into pro‐ or anti‐inflammatory phenotypes based on matrix stiffness

**FIGURE 1:**
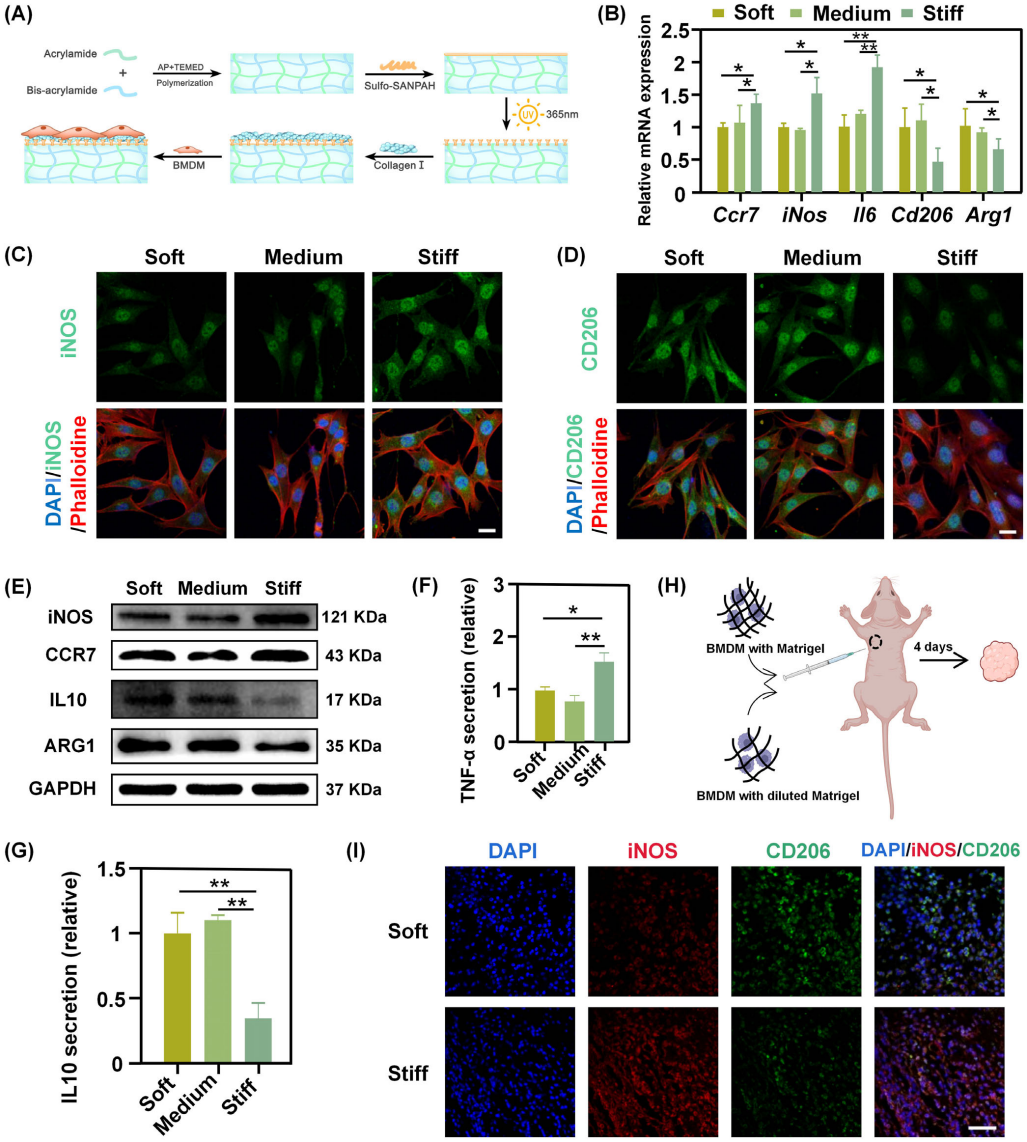
Macrophages polarise into pro‐ or anti‐inflammatory phenotypes based on matrix stiffness. (A) Schematic diagram illustrating the preparation of PA gel substrates. The PA gels were coated with type I collagen at 4°C overnight to facilitate the adhesion of BMDM. AP: Ammonium Persulphate, TEMED: N, N, N0, N0‐tetramethylethylene diamine. (B) The mRNA expression levels of inflammatory genes and anti‐inflammatory genes on soft, medium and stiff PA gels (*p < 0.05; **p < 0.01). (C, D) Representative images of iNOS (C) and CD206 (D) immunofluorescence on soft, medium and stiff substrates (Scale bar: 25 um). (E) Representative WB images of M1/M2 marker proteins on soft, medium and stiff gels. (F, G) TNF‐α (F) and IL10 (G) secretion by BMDM cultured on three matrices of varying stiffness. (H) Schematic illustration of the subcutaneous transplantation of BMDM cells in nude mice, with the original concentration of Matrigel as the stiff group and diluted Matrigel as the soft group. (I) Confocal microscopy images of immunofluorescence staining for the detection of iNOS and CD206 in paraffin sections from the xenograft (Scale bar: 50 μm).

Macrophages are key regulators of immune response homeostasis (pro‐ and anti‐inflammatory) and possess extensive capacity to sense microenvironmental signals and to modulate subsequent immunological response. Macrophages often encounter heterogeneous and complex extracellular matrix (ECM) environments when modulating inflammatory and healing responses after infection or injury.^23^ It has previously been shown that ECM physical characteristics, such as substrate stiffness, may influence macrophage phagocytosis and elasticity.^24^, ^25^ To further understand the effects of ECM stiffness on macrophage function, polyacrylamide (PA) gels with tunable stiffness were employed in our study, which can be achieved by regulating the proportions of acrylamide and N‐N′‐methylene‐bisacrylamide. The entire fabrication protocols of PA gel substrates are illustrated in Figure 1A. BMDM were seeded on collagen‐coated PA gels of varying stiffness, 11 kPa for soft, 88 kPa for medium and 323 kPa for stiff, based on a previous study.^26^ Utilising atomic force microscopy (AFM), we analysed 16 positions of BMDM on soft, medium and stiff substrates and generated the typical mechanics heatmaps. With matrix stiffening, BMDM exhibited an increase in cellular stiffness (Figure S1, Supporting Information). It can thus be inferred that macrophages have extensive capacity to sense and respond to matrix stiffness.

We next explored the matrix stiffness‐induced phenotype of BMDM by analysing the gene and protein expression profiles of BMDM cultured on different gels. PCR showed that the expression levels of M1‐associated gene expression were markedly higher on stiff substrates, as compared to soft and medium substrates, while M2‐associated gene expression presented the opposite trend (Figure 1B). Western blot (WB) also demonstrated similar results. It was found that the expression of C‐C chemokine receptor 7 (CCR7) and inducible nitric oxide synthase (iNOS), canonical markers of M1 macrophages, were significantly up‐regulated in stiff substrates compared to soft substrates. By contrast, canonical markers of M2 macrophages, including arginase‐1 (ARG1) and interleukin10 (IL10), were upregulated upon cultured on soft versus stiff substrates (Figure 1E; Figure S2C). The polarisation state of macrophages on all gels subjected to immunofluorescence staining showed that the phenotype trend pattern was consistent with the results of PCR and western blotting (Figure 1C,D; Figure S2A,B). Moreover, cell culture supernatants of PA gels of varying stiffness were collected for enzyme‐linked immunosorbent assay (ELISA) analyses. BMDM cultured on stiff substrates elicited the highest tumour necrosis factor α (TNF‐α) secretion upon IFN‐γ/LPS activation while yielding the least IL10 secretion upon IL4/IL13 activation (Figure 1F,G). Subsequently, a subcutaneously implanted tumour model in nude mice was utilised to explore the effects of matrix stiffness on BMDM polarisation in vivo (Figure 1H). Briefly, BMDM mixed with Matrigel (stiff group) or diluted Matrigel (soft group) were injected subcutaneously into nude mice. After 4 days, the implanted tissues were collected and stained for iNOS and CD206 by immunofluorescent staining. Consistent with the results *in vitro*, the stiff group showed enhanced iNOS expression, whereas that of CD206 was decreased (Figure 1I; Figure S2D,E). Taken together, these results indicated that macrophages were mechanosensitive to matrix stiffness and adopted corresponding phenotypes according to their surrounding environment. Specifically, macrophages preferably displayed the anti‐inflammatory phenotype (M2) on soft substrates while stiff substrates polarised them towards the pro‐inflammatory phenotype (M1).

### 2.2 Stiffness‐mediated macrophage phenotype is associated with Piezo1 and YAP expression

**FIGURE 2:**
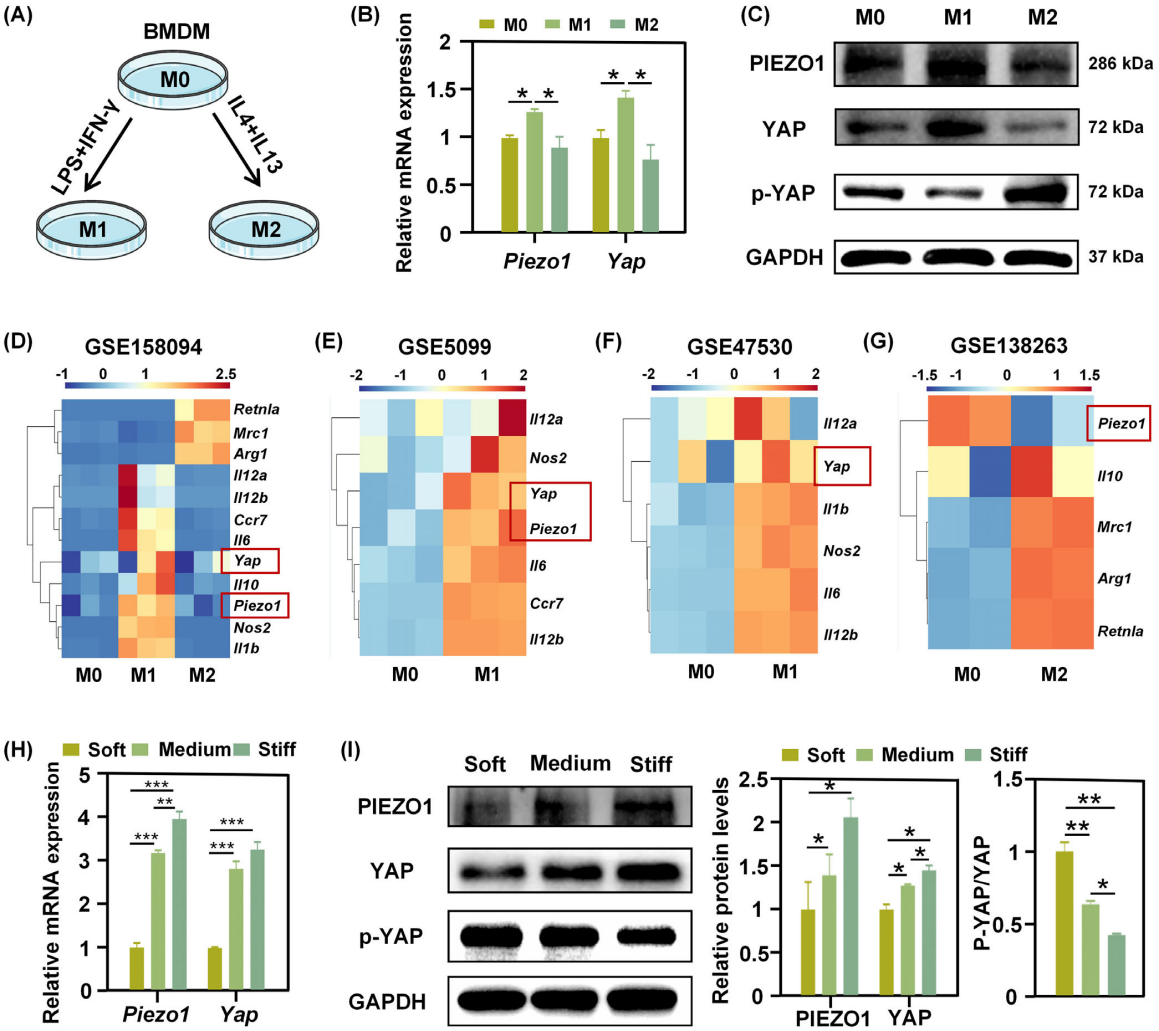
Stiffness‐mediated macrophage phenotype is associated with Piezo1 and YAP expression. (A) Schematic illustration of chemical induction of macrophage differentiation. (B, C) mRNA (B) and protein (C) expression levels of Piezo1 and YAP during M1/M2 differentiation of BMDM (*p < 0.05). (D–G) Gene expression heatmaps of Piezo1, Yap, and canonical marker genes of macrophage M1/M2 phenotypes in public transcriptomic datasets. (H, I) Changes in the expression of Piezo1 and YAP at the mRNA (H) and protein (I) levels on soft, medium, and stiff substrates (**p < 0.01; ***p < 0.001).

Having established that matrix stiffness plays a crucial role in macrophage phenotype, we next explored the possible underlying molecular mechanisms implicated in stress‐mediated macrophage polarisation. Considering the key role of Piezo1 and YAP in mechanotransduction, we examined their expression during M0 macrophage differentiation towards the M1/M2 phenotype. The induction protocol of M0 macrophage differentiation *in vitro* is illustrated in Figure 2A. BMDM were induced to the M1 phenotype with LPS and IFN‐γ (also labelled as M1 conditioned medium) and to the M2 phenotype with IL‐4 and IL‐13 (M2 conditioned medium). The PCR and WB results both indicated that Piezo1 and YAP exhibited similar expression patterns during macrophage differentiation. Specifically, they were up‐regulated during M1 differentiation whereas down‐regulated during M2 differentiation (Figure 2B,C; Figure S3). Subsequently, we collected four transcriptomic datasets related to BMDM polarisation in the GEO database and examined the gene expression patterns of Piezo1, Yap and various canonical marker genes of the M1/M2 phenotypes. The gene expression heatmaps showed that Piezo1 and Yap were upregulated in the M1 phenotype while being downregulated in the M2 phenotype (Figure 2D–G; Figure S4A), which was consistent with the above findings. Furthermore, the correlation heatmaps in Figure S4B‐E exhibited a strong positive correlation between the gene expression trends of Piezo1 and Yap with canonical M1 phenotype marker genes (*Nos2*, *Ccr7*, *Il12b*, *Il12a*, *Il1b*, *Il6*) during macrophage polarisation. Meanwhile, the gene expression trends of Piezo1 and Yap were negatively correlated with M2 phenotype marker genes (*Mrc1*, *Arg1*, *Retnla*, *Il10*) (Figure S4B,F). Together, these results thus demonstrated that the expression of the mechanotransduction effectors Piezo1 and YAP was highly dependent on macrophage polarisation.

Piezo1 and YAP are identified as sensors of mechanical cues within the cellular microenvironment. Matrix stiffening enhances mechanical stimuli being sensed by the cell. Accordingly, the mRNA and protein expression levels of Piezo1 and YAP were successively increased on soft, medium and stiff substrates, and accompanied by increased nuclear YAP localisation (lower p‐YAP/YAP ratio) (Figure 2H,I). This showed that Piezo1 and YAP activity in BMDM were regulated by ECM stiffness. These results, coupled with the expression patterns of these two molecules during macrophage polarisation as described earlier, would thus establish a close association between stiffness‐mediated macrophage M1/M2 phenotypes with Piezo1 and YAP expression.

### 2.3 Activation of Piezo1 promotes M1 macrophage polarisation but suppresses M2 macrophage polarisation

**FIGURE 3:**
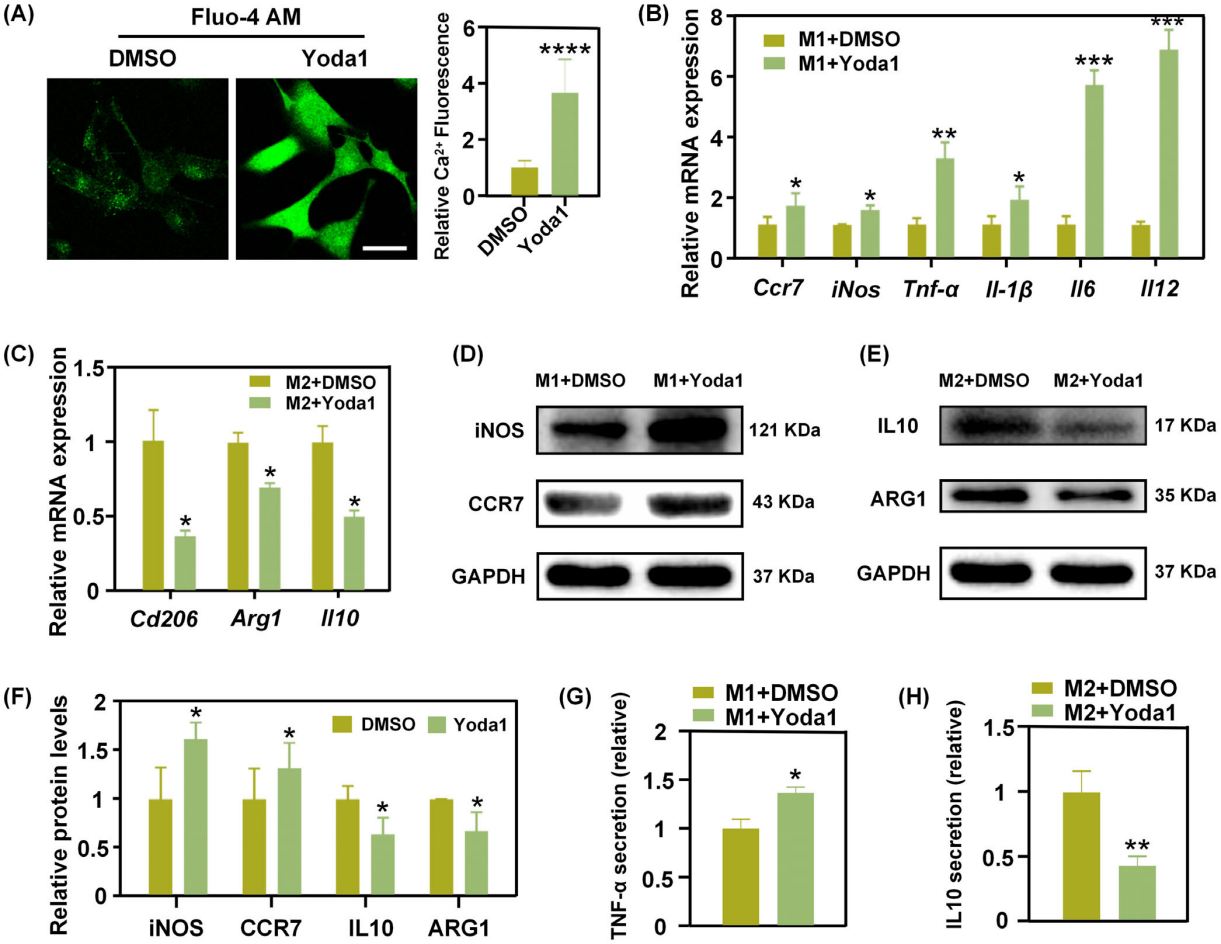
Activation of Piezo1 promotes M1 macrophage polarisation but suppresses M2 macrophage polarisation. (A) Representative fluorescence imaging of intracellular Ca^2+^ in BMDM and the mean fluorescence intensities of Fluo‐4 AM (Scale bar: 30 um) (****p < 0.0001). (B, C) Relative mRNA expression levels of canonical markers of M1 macrophages (B) and M2 macrophages (C) after Yoda1 treatment (*p < 0.05; **p < 0.01, ***p < 0.001). (D–F) Representative WB images of M1 marker proteins (D) and M2 marker proteins (E) after Piezo1 activation. Quantification analysis was displayed in (F). (G, H) Relative TNF‐α (G) and IL10 (H) secretion by BMDM treated with Yoda1.

Generally, the signal triggered by ECM is transmitted through the transmembrane structure. Therefore, we initially investigated the role of Piezo1 in macrophage polarisation. For this purpose, Yoda1, a novel specific agonist of the Piezo1 ion channel was utilised. The Fluo‐4 AM calcium probe revealed that Yoda1 induced calcium influx in BMDM (Figure 3A). In the presence of M1 conditioned medium, Yoda1 increased M1 inflammatory gene expression (Figure 3B). The protein expression levels of M1‐associated markers, CCR7 and iNOS, were also significantly upregulated with Yoda1 treatment (Figure 3D,F). Moreover, the ELISA results also revealed increased secretion of TNF‐α after Yoda1 treatment (Figure 3G). Hence, these data indicated that Piezo1 activation promoted macrophage M1 polarisation. On the other hand, under M2‐inducible conditions, treatment with Yoda1 led to reduced mRNA expression levels of M2‐associated genes (Figure 3C). The M2‐related proteins, ARG1 and IL10, were downregulated by Yoda1 (Figure 3E,F). Additionally, the ELISA results also confirmed that Yoda1 reduced IL10 secretion by BMDM in the M2 conditioned medium (Figure 3H). These data implied that Yoda1 suppressed the polarisation of macrophage towards the M2 phenotype. Taken together, it was clear that Piezo1 ion channels worked synergistically with chemical factors to regulate macrophage polarisation, and that mechanically‐activated Piezo1 favoured macrophage polarisation to the M1 phenotype while suppressing macrophage polarisation to the M2 phenotype.

### 2.4 Silencing of Piezo1 suppresses M1 macrophage polarisation but promotes M2 macrophage polarisation on stiff gels

**FIGURE 4:**
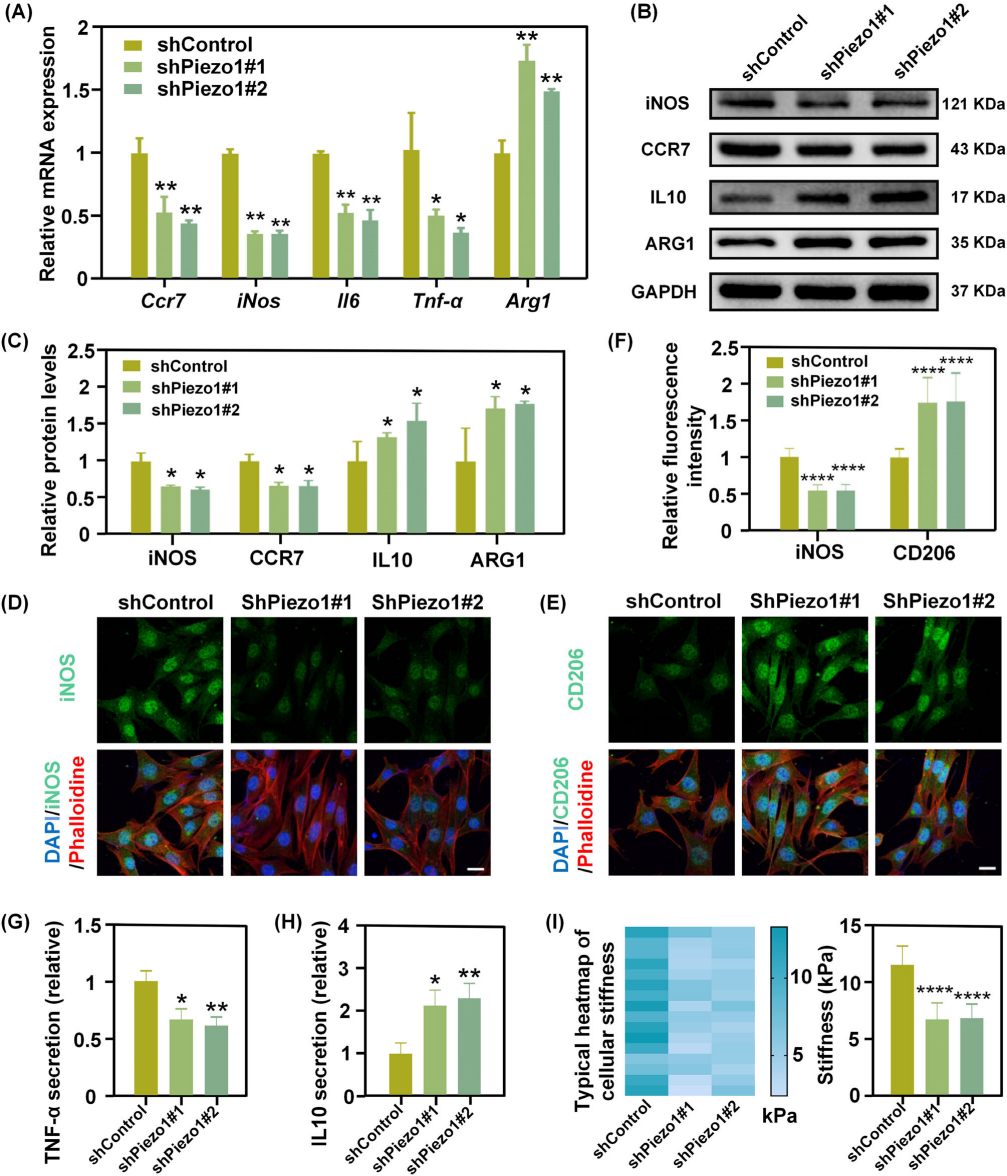
Silencing of Piezo1 suppresses M1 macrophage polarisation but promotes M2 macrophage polarisation on stiff gels. (A) Relative mRNA expression levels of macrophage M1/M2‐associated genes of Piezo1 knockdown and control groups when cultured on stiff substrates (*p < 0.05; **p < 0.01). (B, C) Representative WB images (B) of M1/M2‐associated marker, together with corresponding protein quantification (C). (D‐F) Representative images of iNOS (D) and CD206 (E) immunofluorescence staining of shControl and shPiezo1 cells on stiff PA gels. Quantification of fluorescence intensities was shown in (F) (Scale bar: 25 um) (****p < 0.0001). (G, H) Relative TNF‐α (G) and IL10 (H) secretion by Piezo1 knock‐down or control BMDM cultured on stiff substrates. (I) Representative stiffness heatmaps and quantitative statistics of shControl and shPiezo1 cells on the stiff matrix.

To further probe the role of Piezo1 in regulating stiffness‐mediated macrophage differentiation, we genetically modified BMDM with stable knockdown of Piezo1 using lentiviral transduction of short hairpin RNA (shRNA). The knockdown efficiency of Piezo1 was verified by qPCR (Figure S5A). CCK8 and EdU assays confirmed that Piezo1 knockdown did not affect BMDM proliferation (Figure S5C–E). And Piezo1 knockdown reduced the Ca^2+^ influx induced by Yoda1 (Figure S6). The qPCR results showed that Piezo1 knockdown on stiff substrates suppressed M1‐associated marker gene expression (*Ccr7*, *iNos*, *Il6* and *Tnf‐α*) and instead promoted M2‐associated marker gene expression (*Ym1* and *Arg1*) (Figure 4A). The WB results indicated that, compared with shControl cells, shPiezo1 cells exhibited decreased expression of M1‐associated proteins (iNOS and CCR7) but increased expression of M2‐associated proteins (IL10 and ARG1) when cultured on stiff PA gels (Figure 4B,C). Immunofluorescence staining for detection of M1 marker iNOS and M2 marker CD206 on shControl and shPiezo1 cells cultured on stiff gels also revealed a similar trend (Figure 4D–F). Moreover, silencing of Piezo1 on a stiff matrix led to a decrease in the secretion of the pro‐inflammatory factor TNF‐α but an increase in the anti‐inflammatory factor IL10 by BMDM (Figure 4G,H). Collectively, these data thus confirmed that silencing of Piezo1 on stiff substrates attenuated stiff environment‐induced M1 macrophage polarisation while favouring M2 macrophage polarisation. In addition, cell indentation tests using AFM showed that the Piezo1 knockdown cells on stiff gels were softer than the control, indicating a reduction in mechanical stimuli sensed by BMDM upon knockdown of Piezo1 (Figure 4I).

### 2.5 Piezo1 activates YAP to regulate macrophage polarisation

**FIGURE 5:**
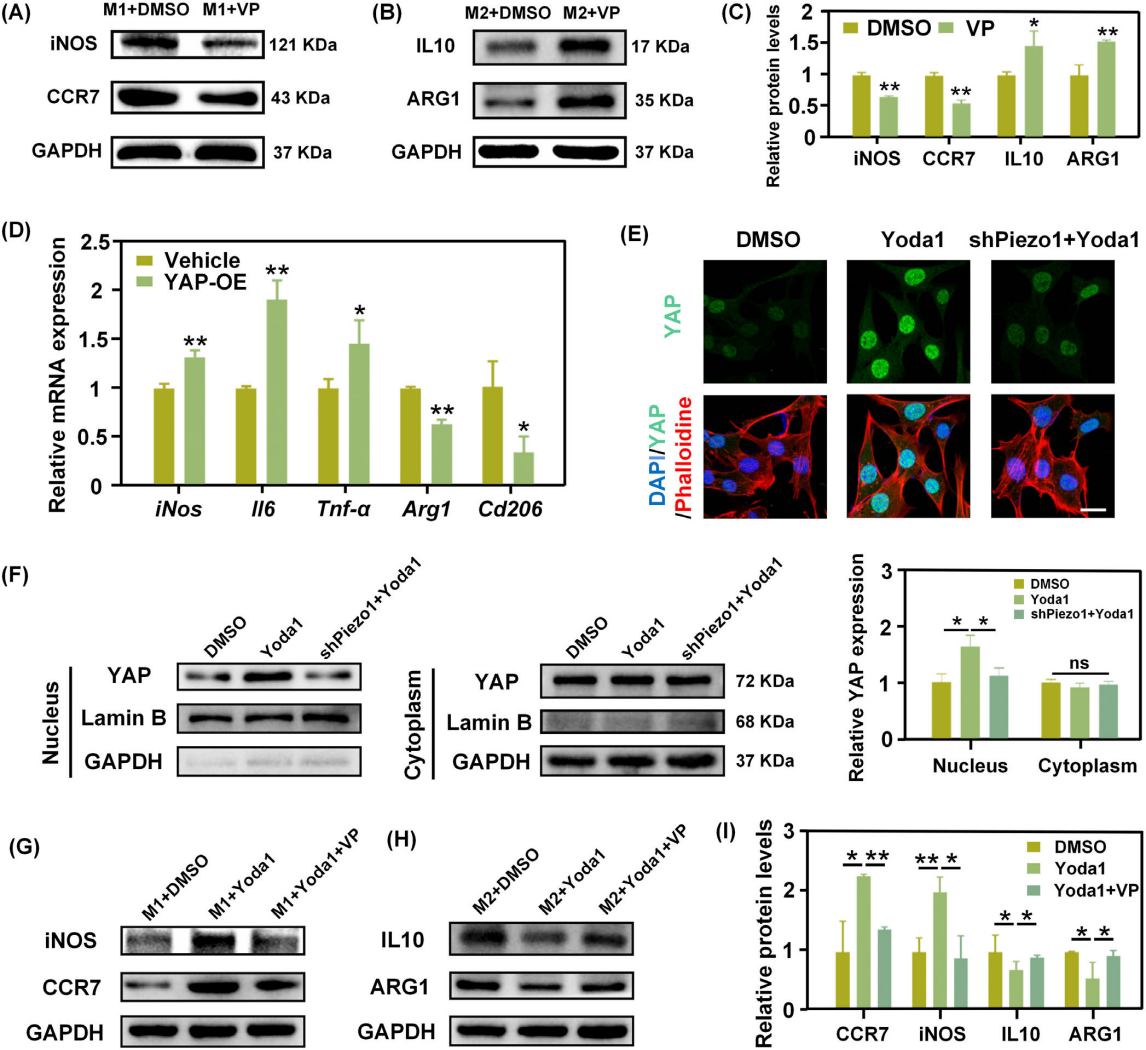
Piezo1 activates YAP to regulate macrophage polarisation. (A, B) Representative WB images of M1‐associated proteins (A) and M2‐associated proteins (B) after VP treatment (*p < 0.05; **p < 0.01). (C) Quantification analysis of protein expression levels in (A) and (B). (D) The expression of pro‐inflammatory and anti‐inflammatory genes when overexpressing YAP. (E) Immunofluorescence staining of YAP (Scale bar: 25 um). (F) Western blotting analysis of YAP subcellular distribution in cytoplasm and nucleus. (G–I) Representative WB images of CCR7, iNOS, IL10 and ARG1 after treatment with Yoda1 alone or Yoda1 + VP. Corresponding quantitative analysis of protein expression levels are shown in (I).

After establishing the role of Piezo1 in regulating stiffness‐mediated macrophage polarisation, we next sought to explore the downstream signalling mechanisms. The Hippo‐YAP signalling pathway is a key regulator of inflammation,^27^ and the preceding results in our study revealed a marked change in YAP expression during M1/M2 polarisation. Hence, we further investigated the role of the Hippo‐YAP signalling pathway in macrophage differentiation. Verteporfin (VP), a YAP inhibitor that inhibits YAP transcriptional activity and function by influencing YAP‐TEAD interactions, was utilised. It was found that VP downregulated M1‐specific protein expression upon M1_(IFN‐γ+LPS)_ induction, while upregulating M2‐specific protein expression upon M2_(IL4+IL13)_ induction (Figure 5A–C). Moreover, BMDM were transfected with plasmid YAP to enable YAP overexpression (Figure S7). The qPCR results showed that YAP overexpression promoted pro‐inflammatory gene expression under M1 induction while inhibiting anti‐inflammatory gene expression under M2 induction (Figure 5D). These findings indicated that YAP worked synergistically with chemical factors to regulate macrophage differentiation, promoting M1 polarisation while inhibiting M2 polarisation. Then, we explored the functional relationship between Piezo1 and YAP in modulating macrophage polarisation. It was observed that the Piezo1 agonist Yoda1 promoted YAP nuclear accumulation, which was significantly inhibited by Piezo1 knockdown (Figure 5E,F). Cells transfected with Piezo1 shRNA displayed a marked reduction in YAP expression compared with non‐targeting shRNA (Figure S5B). The Piezo1 inhibitor GsMTx‐4 significantly reduced YAP nuclear expression by BMDM cultured on stiff gels (Figure S8). It was demonstrated that YAP could be a downstream effector of Piezo1 activity. Subsequently, to further elucidate the up‐ and downstream relationship between Piezo1 and YAP within the context of our research, BMDM were treated with Yoda1 and VP simultaneously. YAP inhibition reversed the effects of enhancing M1 polarisation and inhibiting M2 polarisation induced by Piezo1 activation (Figure 5G–I). YAP activation on stiff substrates also reversed the effects of inhibited M1 polarisation and promoted M2 polarisation induced by Piezo1 inhibitor (Figure S9). Together, these results thus suggest that Piezo1 activate YAP to regulate macrophage polarisation.

### 2.6 YAP deficiency reverses macrophage phenotype in a stiff environment and promotes implant osseointegration

**FIGURE 6:**
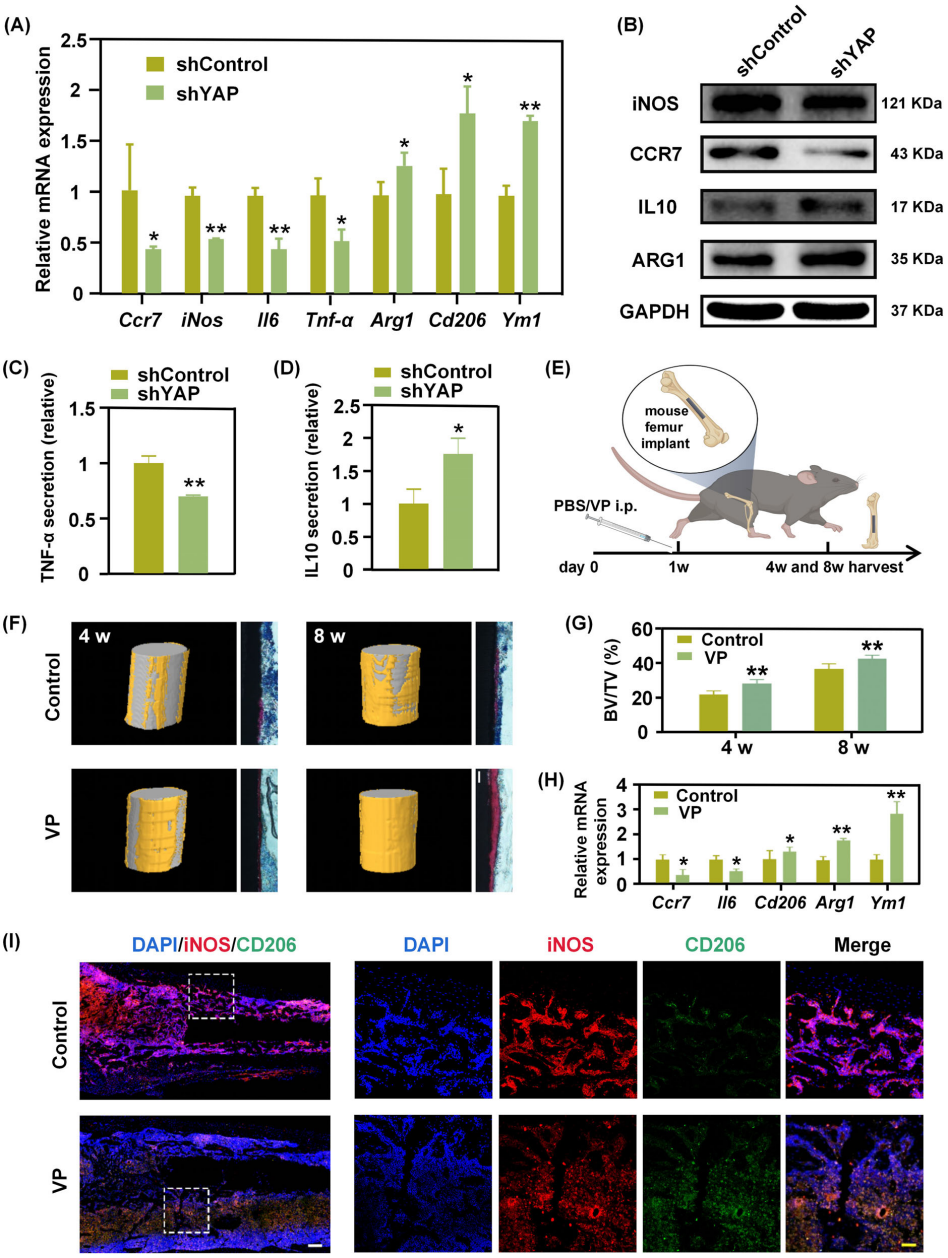
YAP deficiency reverses macrophage phenotype in a stiff environment and promotes implant osseointegration. (A) mRNA expression levels of macrophage M1/M2 marker genes of shControl and shYAP cells on stiff PA gels (*p < 0.05; **p < 0.01). (B) Representative WB images of a canonical marker of M1/M2 macrophages. (C, D) Relative TNF‐α (C) and IL10 (D) secretion by shYAP or the shControl BMDM cultured on stiff substrates. (E) Schematic illustration of the femoral implant model. (F) 3D reconstructed images from micro‐CT test and methylene blue‐acid fuchsin staining of hard tissue slices (Scale bar: 200 um). (G) The calculated bone volume (BV) to total volume (TV) of the control and experimental groups at 4 weeks and 8 weeks post‐implantation. (H) Relative mRNA expression levels of M1 and M2 marker genes at 2 weeks post‐implantation. (I) Representative images of femoral bone tissue sections subjected to immunofluorescence staining for detection of iNOS and CD206 at 2 weeks post‐implantation (Scale bar: left 200 um, right 50 μm).

We next determined whether YAP is responsible for stiffness‐mediated macrophage phenotype. To this end, YAP expression in BMDM was knocked down by shRNA lentivirus transfection. Successful transfection was verified by PCR (Figure S10A). Results of the EdU assay confirmed that silencing of YAP did not affect cell proliferation (Figure S10B). PCR and WB assays showed that YAP‐knockdown BMDM seeded on stiff substrates inhibited M1‐associated marker expression, while promoting M2‐associated marker expression (Figure 6A,B; Figure S11A). Furthermore, ELISA revealed decreased TNF‐α secretion and increased IL10 secretion of shYAP cells cultured on stiff gels, as compared with the control group (Figure 6C,D). Collectively, these results thus confirmed that silencing of YAP on stiff substrates suppressed the pro‐inflammatory M1 phenotype while favouring the anti‐inflammatory M2 phenotype. In addition, AFM showed a reduction in cellular stiffness on stiff PA gels after YAP knockdown (Figure S12), which thus suggested reduced mechano‐perception by shYAP cells that may be associated with altered mechanical properties of the cytoskeleton.

Macrophages play a pivotal role in regulating the cascade of immunological responses towards implants. Successful implant integration depends on achieving a delicate equilibrium between M1 and M2 macrophages.^28^, ^29^ Sustained and elevated M1 response can result in chronic inflammation, accompanied by fusion of macrophages, formation of foreign‐body giant cells, and breakdown of healthy tissues surrounding the implant, eventually leading to implant failure.^30^ Therefore, manipulating macrophage polarisation towards the M2 phenotype may improve the outcome of implant application. Because YAP plays a pivotal role in regulating macrophage fate within a stiff environment, we next investigated whether YAP inhibition can exert positive effects on Ti implantation. A model of Ti nail implantation into the femoral bone was used, and the entire flowchart of the animal experiment was illustrated in Figure 6E. New bone formation around the implant was characterised using 3D micro‐computed tomography and hard‐tissue histological sections. We found that applying the YAP inhibitor verteporfin during the early stage (the first week) of implant placement enhanced implant osseointegration at both 4 weeks and 8 weeks post‐surgery (Figure 6F,G). Immunofluorescence staining showed significantly enhanced levels of osteopontin expression in the VP group at 2 weeks post‐surgery (Figure S13). PCR demonstrated decreased mRNA levels of *Ccr7* and *Il6*, with elevated levels of *Cd206* and *Arg1* in the VP group at 2 weeks post‐implantation (Figure 6H). Moreover, immunofluorescence staining of iNOS and CD206 from femoral tissues showed that the YAP inhibitor reduced the M1/M2 ratio of macrophages surrounding the implant (Figure 6I; Figure S11B,C). In summary, the above results verified that YAP inhibition can reverse the polarisation of macrophage towards the anti‐inflammatory M2 phenotype within a stiff environment, thereby promoting bone‐implant integration. We successfully mitigated implant‐induced inflammation through manipulation of the macrophage phenotype, and improved implant acceptance by the host tissue.

## 3 DISCUSSION

Macrophages are one of the first responders of the immune system upon biomaterial implantation, followed by the release of chemokines that recruit additional macrophages and other immune cells.^31^ In recent years, there has been increasing awareness regarding the importance of macrophages in the healing of implant‐related complications within the biomaterial community. Macrophages can rapidly modulate their behaviour in response to local cues.^32^ Previous studies have addressed the effects of substrate stiffness on macrophage function. It was shown that macrophage phagocytosis increased with the stiffness of PA gels (2 and 150 kPa).^12^ Consistent with our findings, Blakney et al. found that LPS‐stimulated macrophages displayed an increase in inflammatory cytokine production with matrix stiffening (130, 240 and 840 kPa).^33^ Previtera et al. also confirmed that macrophages grown on soft substrates produced less pro‐inflammatory mediators when grown on stiff substrates upon stimulation with TNF‐α (0.3–230 kPa).^25^ However, the precise mechanisms of how matrix stiffness drives the pro‐inflammatory responses of macrophages are not well understood. Moreover, the effect of matrix stiffness on macrophage anti‐inflammatory phenotype has not yet been investigated. Macrophages encounter a whole range of tissue stiffness *in vivo*, ranging from a few hundred pascals in fat tissues, 30 kPa for muscles, and up to 300 kPa in atherosclerotic plaques.^34^, ^35^, ^36^ The stiffness value (10–323 kPa) of the PA gels we used represents a wide range of stiffness encountered in both healthy and diseased tissues. Moreover, our work provides a clearer and more comprehensive picture of the ‘priming’ of macrophages towards the pro‐ (M1) and anti‐inflammatory (M2) states, as well as provides a potential molecular mechanism to explain such regulation mediated by matrix stiffness.

**FIGURE 7:**
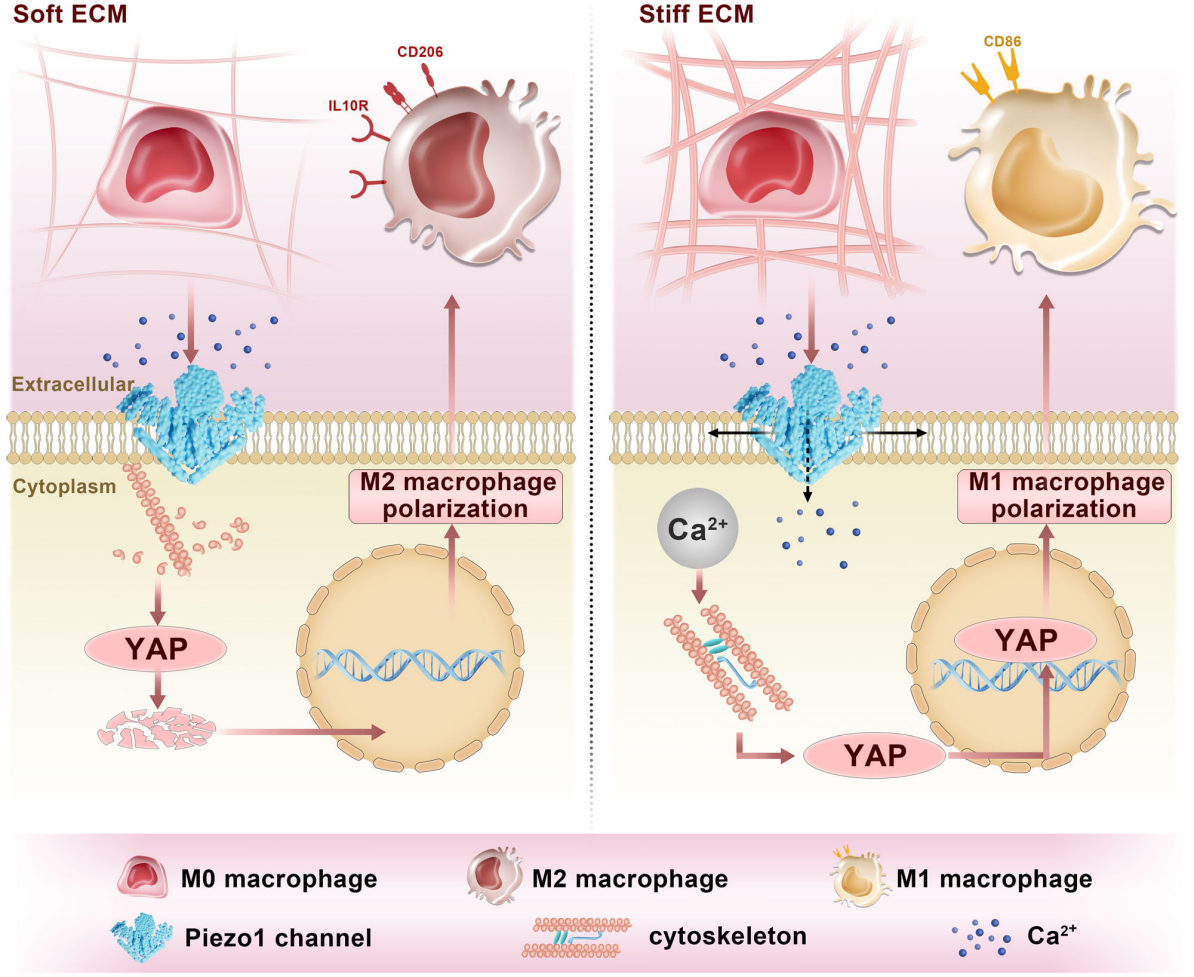
Schematic diagram illustrating the underlying mechanisms by which matrix stiffness regulates macrophage polarisation. On stiff ECM, Piezo1‐YAP signalling‐induced mechanotransduction promotes macrophage differentiation towards the M1 phenotype. On the other hand, inhibition of the Piezo1‐YAP signalling axis on soft ECM facilitates macrophage differentiation towards the M2 phenotype.

Here in this study, we propose that matrix stiffness affects the function of bone marrow‐derived macrophages, inducing an anti‐inflammatory M2 phenotype on soft and medium stiffness substrates and a pro‐inflammatory M1 phenotype on stiff substrates. In terms of molecular mechanisms, we have identified the pivotal role of two signalling molecules, Piezo1 and YAP, in stiffness‐mediated macrophage polarisation. Stiff ECM activates Piezo1 channels and promotes Ca^2+^ influx, resulting in cytoskeleton reorganisation. Subsequently, increased tension within the actin cytoskeleton facilitates YAP expression and nuclear translocation, ultimately linking mechanosensing to nuclear transcription factor activity and promoting macrophage polarisation to the M1 phenotype (Figure 7). Conversely, the overall mechanosensing is weak on soft ECM, leading to little or no activation of the Piezo1‐YAP signalling axis, which therefore promotes macrophage polarisation to the M2 phenotype. Finally, we successfully induced repolarisation of macrophages towards the M2 state within a stiff microenvironment at the implant site and achieved the goal of promoting implant osseointegration via treatment with a YAP inhibitor. This study identifies new therapeutic targets for the promotion of bone formation in implants and provides cues for the design of immuno‐responsive biomaterials.

Nevertheless, some limitations of our research must be acknowledged. First, the stiffness values of our experimental groups were relatively limited in number. In the future, it would be preferable to generate a greater number of experimental groups with varying stiffness, thereby representing the gradient of stiffness changes and its relationship with macrophage functionality. Additionally, despite there being growing evidence supporting the notion that transcription factor activity is mechanically regulated, we have not yet conducted further investigations to delineate how YAP nuclear translocation regulates macrophage differentiation.

## 4 CONCLUSION

In this study, we reported that macrophages adopt the pro‐inflammatory M1 phenotype in response to rigid substrates while polarising towards the anti‐inflammatory M2 phenotype in a soft environment. Mechanically, a stiff matrix activates the Piezo1‐YAP signalling axis in macrophages to promote M1 polarisation, which is inhibited on a soft matrix. YAP can potentially be a therapeutic target for enhancing osseointegration of implants. Elevated stiffness has been correlated with increased inflammatory activation in cardiovascular diseases. This study, on the one hand, provides novel treatment strategies for some pathological conditions, while on the other hand, facilitates immuno‐modulatory biomaterial design.

## 5 EXPERIMENTAL SECTION

### 5.1 Cell culture

Mouse BMDM were isolated and cultured as previously described.^37^ The cells were cultured in a high‐glucose DMEM medium at 37°C within a 5% CO_2_ incubator.

### 5.2 Cell transfection

Lentivirus was produced using 293 T cells.

Sequence of Piezo1 shRNA #1:

5′‐CTGCTATCAGACACCATTTAT‐3′.

Sequence of Piezo1 shRNA #2:

5′‐GAATCCTGCTGCTGCTATATT‐3′.

Sequence of YAP shRNA:

5′‐GCGGTTGAAACAACAGGAATT‐3′.

For lentivirus transfection *in vitro*. BMDM were seeded and cultured until 80% confluence, followed by transfection with lentivirus (fresh medium: filtered medium containing virus = 1: 1) for 48 h. Stable transfected cells were obtained through puromycin selection for 1 week.

For the overexpression of YAP, the YAP overexpression plasmid was purchased from SinoBiological (China).

### 5.3 Polarisation of BMDM

For the differentiation of macrophages, the M1 conditioned medium was supplemented with 5 ng/mL interferon‐γ (IFN‐γ) and 100 ng/mL lipopolysaccharide (LPS), and M2 conditioned medium was supplemented with 20 ng/mL of interleukin‐4 and interleukin‐13 (IL‐4 and IL‐13). Cells were induced for 24 h before harvesting.

### 5.4 RT‐qPCR analysis

The total RNA from BMDM was extracted using Trizol reagent (Invitrogen), and reverse transcribed to cDNA using a PCR thermal cycler (Takara). Then, optical 96‐well reaction plates and optical adhesive films (Thermo Fisher Scientific) were used for PCR. The relative gene expression was quantified using the 2^⁻ΔΔCt^ method. The primer sequences are listed in Table S1. GAPDH served as the internal housekeeping control gene.

### 5.5 Western blot

Total cellular protein was extracted using a protein extraction buffer. The cell lysate protein concentration was determined by a BCA protein assay kit (Pierce, Thermo Fisher Scientific). Before electrophoresis, protein samples were denatured in Laemmli buffer at 95°C for 10 min. Then, these were subjected to SDS‐PAGE and transferred by electroblotting to PVDF membranes (Millipore, IPVH00010). The membranes were then blocked in 5% (w/v) low‐fat milk in TBST buffer and incubated with the following primary antibodies: anti‐GAPDH (10494‐1‐AP; Proteintech), anti‐Piezo1 (15939‐1‐AP; Proteintech), anti‐YAP (14074S; Cell Signalling Technology), anti‐p‐YAP (4911S; Cell Signalling Technology) anti‐Lamin B (12987‐1‐AP; Proteintech), anti‐iNOS (340668; Zen BioScience), anti‐CCR7 (380950; Zen BioScience), anti‐IL‐10 (12163S; Cell Signalling Technology), and anti‐Arginase1 (ab91279; Abcam) overnight at 4°C. Species‐matched peroxidase‐conjugated secondary antibodies were incubated for 1 h at room temperature and visualised by chemiluminescence. The grey pixel values of the protein bands were analysed by Image J software.

### 5.6 Protein secretion analysis using ELISA

The release of cytokines by BMDM cultured on the gels of varying stiffness was quantified using ELISA. The concentrations of tumour necrosis factor‐α (TNF‐α) (abs520010) and interleukin‐10 (IL‐10) (abs520005) were quantified by ELISA using respective kits following the manufacturer's instructions (Absin). All samples were collected in duplicates.

### 5.7 Atomic force microscopy

In this study, AFM was used to evaluate the stiffness of BMDM cultured on PA gels. AFM experiments were carried out under the operation mode of PeakForce QNM in Fluid available (Bruker, Billerica, MA, USA). Force mappings were obtained using a silicon nitride probe (PFQNM‐LC‐A‐CAL, Bruker). Force curves were captured by Force Volume mode with a scan size of 500 nm. To obtain the apparent Young's modulus, all data were analysed with the Sneddon model using Nanoscope Analysis software (Bruker).

### 5.8 Preparation of PA gels

We prepared PA gels with different stiffness by mixing different concentrations of acrylamide (Acr) and bis‐acrylamide (Bis‐Acr). Soft substrates were made of 6% (w/v) Acr and 0.15% (w/v) Bis‐Acr, medium stiffness substrates were made of 10% (w/v) Acr and 0.3% (w/v) Bis‐Acr, while stiff substrates were made of 20% Acr (w/v) and 0.8% (w/v) Bis‐Acr, polymerised in the presence of N, N, N0, N0‐tetramethylethylene diamine, and ammonium persulphate. Next, gels were allowed to polymerise for 30 min and immersed in 10 mM HEPES buffer containing 2% (v/v) penicillin/streptomycin solution for at least 48 h. These were then treated with 0.2 mg/mL Sulfosuccinimidyl 6‐(40‐azido‐20‐nitrophenylamino) hexanoate (Sulfo‐SANPAH) (Sigma) for 20 min in the presence of long wave UV (365 nm). After washing with 50 mM HEPES buffer, gels were incubated with 0.2 mg/mL collagen type I (BD BioSciences) overnight at 4°C and washed three times in phosphate buffered saline (PBS) before cell seeding. All steps were performed on a UV‐sterilised ultra‐clean table and aseptic techniques were employed.

### 5.9 Fluorescent immunohistochemistry

Tissue processing and sectioning were carried out as previously described.^38^ Images were captured using confocal microscopy (Leica). The fluorescence intensity was quantified using the Image J software.

### 5.10 Public transcriptomic data collection and analysis

Four transcriptomic datasets related to BMDM polarisation were collected from the GEO database (https://www.ncbi.nlm.nih.gov/geo/). The dataset accessions are GSE158094 (RNA‐seq dataset), GSE5099, GSE47530 and GSE138263 (microarray datasets). For each dataset, the normalised whole‐genome gene expression matrix was downloaded from the GEO database. Then the gene expression matrix of Piezo1, Yap and canonical marker genes of M1/M2 phenotypes were extracted according to gene ID or microarray probe set ID. The pheatmap function in the R package pheatmap (version 1.0.12) was used to visualise the gene expression patterns. The sparcc function in R package fastspar (version 0.0.10) and the hclust function in R package stats (version 3.6.3) were used to calculate the spearman correlation coefficients and generate the correlation heatmaps.

### 5.11 Agonists and inhibitors

Piezo1 agonist Yoda1 (5 μM), Piezo1 inhibitor GsMTx4 (1 μM), YAP inhibitor verteporfin (VP, 2 μM), and YAP agonist PY‐60 (10 μM) were all purchased from Medchemexpress, New Jersey, America. To determine the effects of Piezo1 or YAP on macrophage polarisation, BMDM were treated with Yoda1 or VP for 24 h. To detect YAP expression, Yoda1 treatment was carried out for 1 h. When calcium imaging was performed, Yoda1 treatment was carried out for 30 min. For the combined application of Yoda1 and VP, BMDM were first treated with Yoda1 for 12 h and then treated with VP for 12 h. For the combined application of GsMTx4 and PY‐60, BMDM were first treated with GsMTx4 for 12 h and then treated with PY‐60 for 12 h.

### 5.12 Ethics statement

All procedures were performed according to established ethical guidelines and approved by the Institutional Animal Care and Use Committee of Peking University (Approval number: LA2023336). The mice were purchased from Beijing HFK Bioscience Co. Ltd. All efforts were made to minimise the suffering of animals.

### 5.13 Animals and surgical procedures

For the implant model, healthy 8‐week‐old male C57BL/6J mice (*n* = 6) were anaesthetised with 1% (w/v) sodium pentobarbital solution. The skin covering the knee was incised, and a scalpel was used to longitudinally divide the patellar tendon, which was then retracted to expose the femoral condyles. A dental bur was used to access the medullary canal, and the implant was placed into the cavities along the longitudinal axis of the femur. After surgery, the experimental group mice were given intraperitoneal (i.p.) injections of verteporfin (VP, 25 mg/kg) once a day for a week, while the control group received PBS at an equal volume. Mice were sacrificed at 2, 4 and 8 weeks post‐surgery, and femurs containing implants were harvested for CT scans and subsequent staining.

For the subcutaneous transplantation model, 3‐week‐old male nude mice (*n* = 5) were injected subcutaneously with 10^7^ BMDM mixed with 0.1 mL Matrigel or diluted Matrigel (Matrigel: PBS = 1: 1). Implants were harvested after 4 days and then fixed with 4% (w/v) paraformaldehyde and paraffin‐embedded for subsequent staining.

### 5.14 Preparation of implant samples

Titanium cylindrical implants (0.6 mm in diameter and 4 mm in length) were custom‐made of TA4 pure titanium, and sandblasted by coarse sand (0.5 mm). Titanium implants were then treated with SLA surface modifications before *in vivo* implantation.

### 5.15 Micro‐CT tests

In total, six samples of each group were scanned at 4 and 8 weeks post‐surgery using a computed micro‐tomography x‐ray (micro‐CT) (Bruker). Serial two‐dimensional cross‐sections were assembled into 3D reconstructions and analysed using the SkyScan software and CTAn. Bone‐to‐implant contact was quantified within a tubular region of interest (ROI) at the distal portion of each femur and calculated as BV/TV (bone volume/total volume) values. After 3D visualisation, ROI regions were marked with pseudo colours.

### 5.16 Statistical analysis

All results were expressed as the mean ± standard error of the mean (SEM). Comparisons between the two groups were performed with the unpaired Student's *t*‐test (two‐tailed). For comparisons of multiple groups, analysis of variance (ANOVA) with Fisher's least significant difference test was used. *p*‐Values less than 0.05 (*p* < 0.05) were considered statistically significant.

## AUTHOR CONTRIBUTIONS

**Feng Mei**: Investigation; methodology; data analysis; writing—original draft. **Yaru Guo**: Investigation; methodology; writing—original draft; funding acquisition. **Yu Wang**: Investigation; methodology; data analysis; writing—original draft. **Yingying Zhou, Boon Chin Heng**: Methodology; writing—review & editing. **Mengru Xie, Xiaofei Huang**: Methodology; writing—original draft; **Shihan Zhang, Shuai Ding, Fangyong Liu**: Investigation; data analysis. **Xuliang Deng, Lili Chen, Cheng Yang**: Conceptualisation; supervision; funding acquisition; writing—review & editing. All authors take part in the discussion.

## CONFLICT OF INTEREST STATEMENT

The authors have no conflicts of interest to declare.

## Supporting information


**FIGURE S1.** The stiffness of BMDM on soft, medium and stiff substrates was measured using atomic force microscopy, and representative cellular stiffness heatmaps on each substrate were displayed on the left (****p < 0.0001).
**FIGURE S2.** (A, B) Quantitative fluorescence intensities of iNOS (A) and CD206 (B) on soft, medium and stiff substrates (****p < 0.0001). (C) Quantitative results of Figure 1E (*p < 0.05; **p < 0.01). (D, E) iNOS (D) and CD206 (E) fluorescence intensity quantification of Figure 1I.
**FIGURE S3.** Quantitative analysis of protein expression levels in Figure 2C (*p < 0.05, **p < 0.01).
**FIGURE S4.** (A) Gene expression heatmap of Piezo1, Yap and canonical marker genes of macrophage M1 phenotype in microarray dataset GSE138263. (B) Correlation heatmap of Piezo1, Yap and canonical marker genes of macrophage M1/M2 phenotypes in RNA‐seq dataset GSE158094. (C–E) Correlation heatmap of Piezo1, Yap and canonical marker genes of macrophage M1 phenotype in microarray datasets GSE5099, GSE47530 and GSE138263. (F) Correlation heatmap of Piezo1 and canonical marker genes of macrophage M2 phenotype in microarray dataset GSE138263.
**FIGURE S5.** (A) Piezo1 knockdown was verified by RT‐PCR (****p < 0.0001). (B) Downregulation of YAP mRNA expression after knockdown of Piezo1 (*p < 0.05). (C–E) The proliferation capacity of shControl and shPiezo1 cells was evaluated using CCK8 (C) and EdU assays (D, E) (Scale bar: 75 μm).
**FIGURE S6.** Representative fluorescence images of Fluo‐4 AM‐loaded BMDM and the quantitative fluorescence intensity of Fluo‐4 AM (Scale bar: 30 um) (****p < 0.0001).
**FIGURE S7.** The transfection efficiency of the YAP overexpression plasmid was verified by RT‐PCR (**p < 0.01).
**FIGURE S8.** YAP immunofluorescence staining after GsMTx‐4 treatment on the stiff PA gels (Scale bar: 20 um) (***p < 0.001).
**FIGURE S9.** Relative mRNA expression levels of M1/M2‐associated genes after DMSO, GsMTx4, GsMTx4 + PY‐60 treatment when cultured on stiff substrates (*p < 0.05; **p < 0.01).
**FIGURE S10.** (A) Validation of YAP knockdown (****p < 0.0001). (B) EdU assay was performed to evaluate the proliferative potentials of shControl and shYAP BMDM (Scale bar: 75 um).
**FIGURE S11.** (A) Quantitative analysis of the protein expression in Figure 6B (*p < 0.05; **p < 0.01). (B, C) The statistical analysis of fluorescence intensities of iNOS (B) and CD206 (C) in Figure 6I (****p < 0.0001).
**FIGURE S12.** Quantitative statistics of stiffness of the shControl and shYAP BMDM cultured on stiff substrates, together with representative heatmaps (****p < 0.0001).
**FIGURE S13.** Representative images of immunofluorescence staining for osteopontin in femoral bone tissue and quantitative analysis of fluorescence intensity (Scale bar: 50 um) (****p < 0.0001).
**TABLE S1.** Primer sequences used for quantitative real‐time PCR analysis.

## Data Availability

All original data can be obtained from the authors upon reasonable request.
