# Supplementary material for: Matrix stiffness regulates macrophage polarisation via the Piezo1‐YAP signalling axis
Source: Cell Prolif. 2024 Mar 31;57(8):e13640. doi: 10.1111/cpr.13640 (PMC11294424; doi:10.1111/cpr.13640)
Supplement: Supplementary file 1 — FIGURE S1. The stiffness of BMDM on soft, medium and stiff substrates was measured using atomic force microscopy, and representative cellular stiffness heatmaps on each substrate were displayed on the left (****p < 0.0001). FIGURE S2. (A, B) Quantitative fluorescence intensities of iNOS (A) and CD206 (B) on soft, medium and stiff substrates (****p < 0.0001). (C) Quantitative results of Figure 1E (*p < 0.05; **p < 0.01). (D, E) iNOS (D) and CD206 (E) fluorescence intensity quantification of Figure 1I. FIGURE S3. Quantitative analysis of protein expression levels in Figure 2C (*p < 0.05, **p < 0.01). FIGURE S4. (A) Gene expression heatmap of Piezo1, Yap and canonical marker genes of macrophage M1 phenotype in microarray dataset GSE138263. (B) Correlation heatmap of Piezo1, Yap and canonical marker genes of macrophage M1/M2 phenotypes in RNA‐seq dataset GSE158094. (C–E) Correlation heatmap of Piezo1, Yap and canonical marker genes of macrophage M1 phenotype in microarray datasets GSE5099, GSE47530 and GSE138263. (F) Correlation heatmap of Piezo1 and canonical marker genes of macrophage M2 phenotype in microarray dataset GSE138263. FIGURE S5. (A) Piezo1 knockdown was verified by RT‐PCR (****p < 0.0001). (B) Downregulation of YAP mRNA expression after knockdown of Piezo1 (*p < 0.05). (C–E) The proliferation capacity of shControl and shPiezo1 cells was evaluated using CCK8 (C) and EdU assays (D, E) (Scale bar: 75 μm). FIGURE S6. Representative fluorescence images of Fluo‐4 AM‐loaded BMDM and the quantitative fluorescence intensity of Fluo‐4 AM (Scale bar: 30 um) (****p < 0.0001). FIGURE S7. The transfection efficiency of the YAP overexpression plasmid was verified by RT‐PCR (**p < 0.01). FIGURE S8. YAP immunofluorescence staining after GsMTx‐4 treatment on the stiff PA gels (Scale bar: 20 um) (***p < 0.001). FIGURE S9. Relative mRNA expression levels of M1/M2‐associated genes after DMSO, GsMTx4, GsMTx4 + PY‐60 treatment when cultured on stiff substrates (*p < 0.05; **p < [file CPR-57-e13640-s001.docx]

Supplementary Materials for

Matrix stiffness regulates macrophage polarization via the Piezo1-YAP signaling axis

*Feng Mei*^1,2,3^, *Yaru Guo*^4^, *Yu Wang*^5^, *Yingying Zhou*^6^, *Boon Chin Heng*^7^, *Mengru Xie*^1,2,3^,

*Xiaofei Huang*^1,2,3^, *Shihan Zhang*^4^, *Shuai Ding*^5^, *Fangyong Liu*^6^

*Xuliang Deng*^4,6^*, *Lili Chen*^1,2,3^*, *Cheng Yang*^1,2,3^*

^1^Department of Stomatology, Union Hospital, Tongji Medical College, Huazhong University of Science and Technology, Wuhan 430022, China

^2^School of Stomatology, Tongji Medical College, Huazhong University of Science and Technology, Wuhan 430022, China

^3^Hubei Province Key Laboratory of Oral and Maxillofacial Development and Regeneration, Wuhan 430022, China

^4^Department of Geriatric Dentistry, Peking University School and Hospital of Stomatology, Beijing 100081, China

^5^Department of Orthodontics, Peking University School and Hospital of Stomatology, National Clinical Research Center for Oral Diseases & National Engineering Laboratory for Digital and Material Technology of Stomatology & Beijing Key Laboratory of Digital Stomatology, Beijing 100081, China

^6^NMPA Key Laboratory for Dental Materials, Department of Dental Materials & Dental Medical Devices Testing Center, Peking University School and Hospital of Stomatology, Beijing 100081, China

^7^Central Laboratory, Peking University School and Hospital of Stomatology, Beijing 100081, China

**This file includes:**

Figure S1 to S13

Table S1.


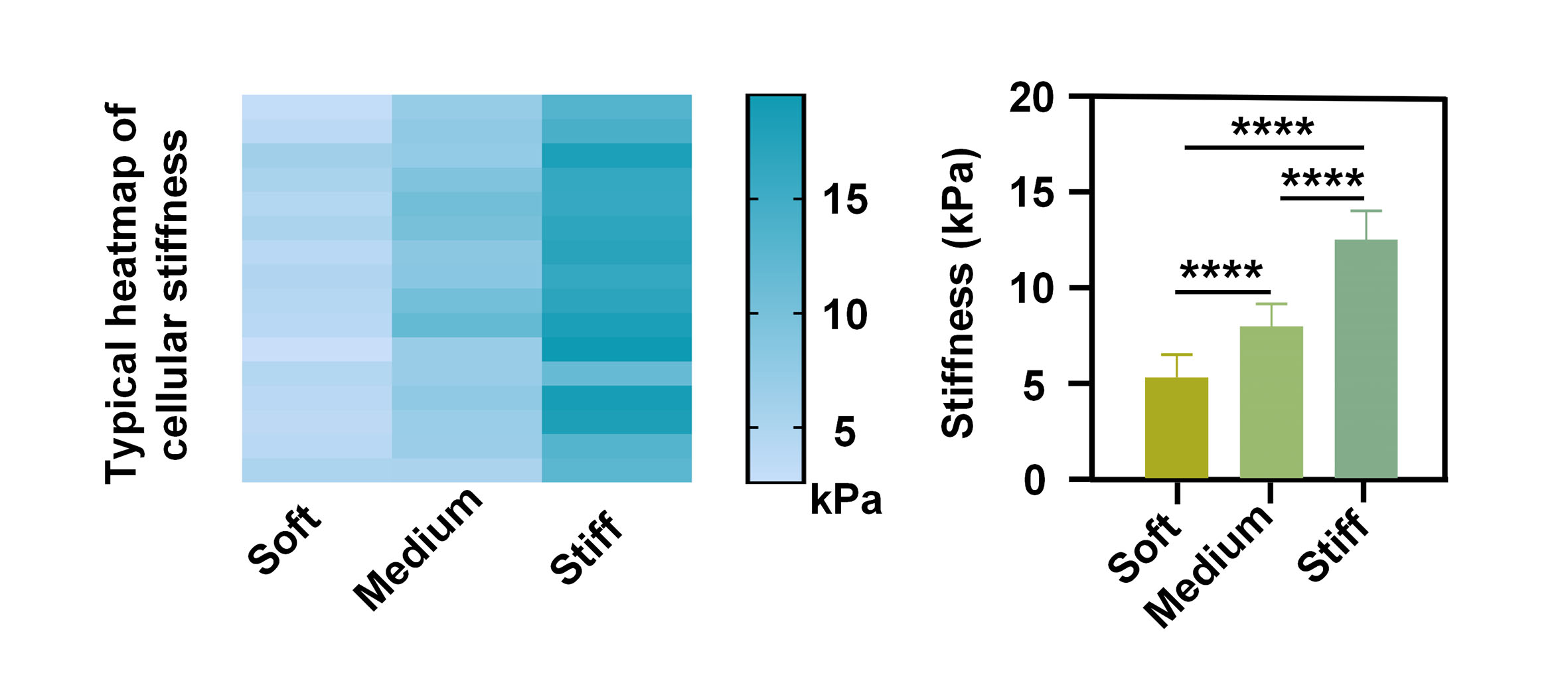


**Figure S1.** The stiffness of BMDM on soft, medium, and stiff substrates was measured using atomic force microscopy, and representative cellular stiffness heatmaps on each substrate were displayed on the left (****P<0.0001).


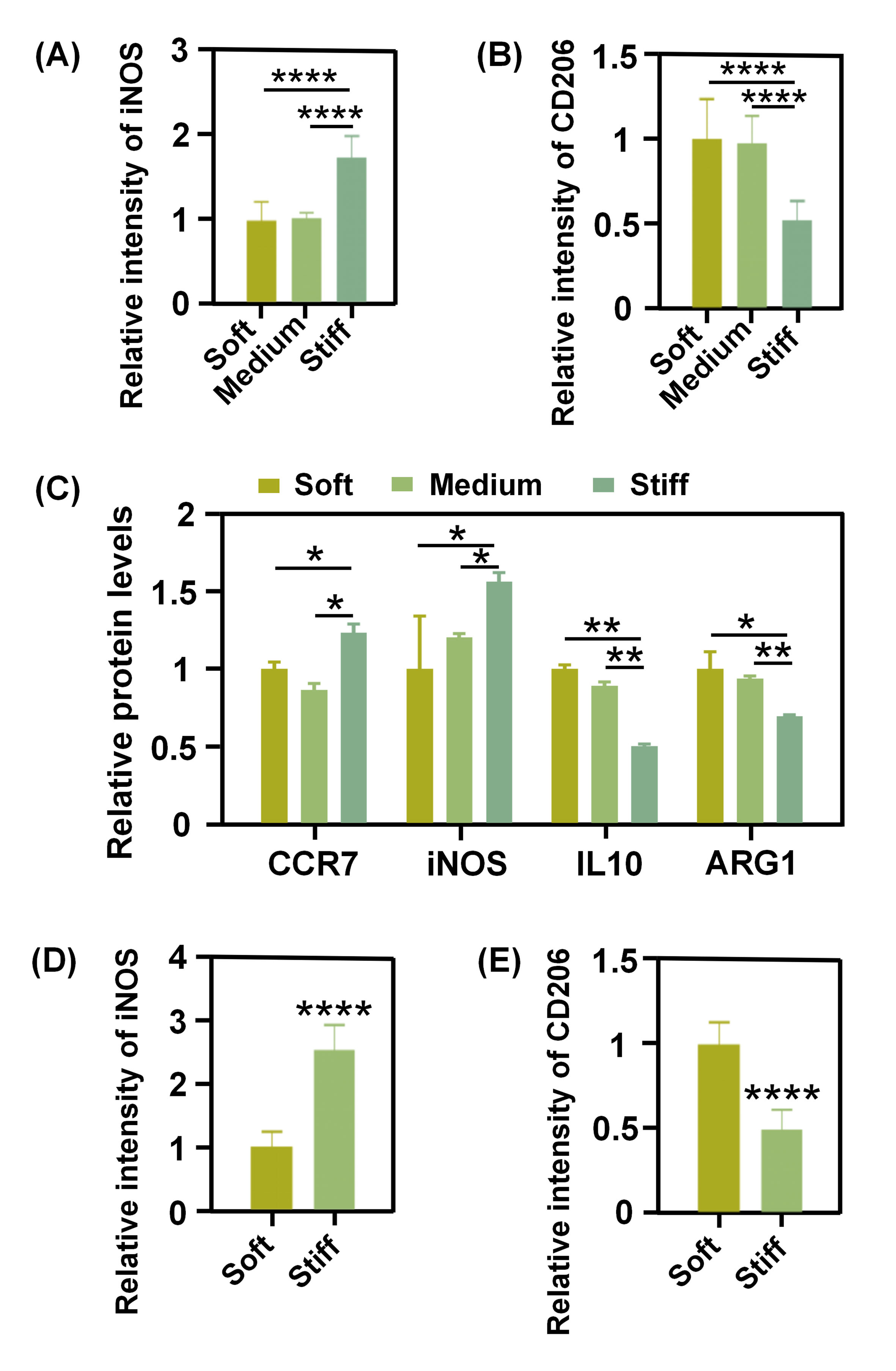


**Figure S2.** (A-B) Quantitative fluorescence intensities of iNOS (A) and CD206 (B) on soft, medium, and stiff substrates (****P<0.0001). (C) Quantitative results of Figure 1E (*P<0.05, **P<0.01). (D-E) iNOS (D) and CD206 (E) fluorescence intensity quantification of Figure 1I.

**
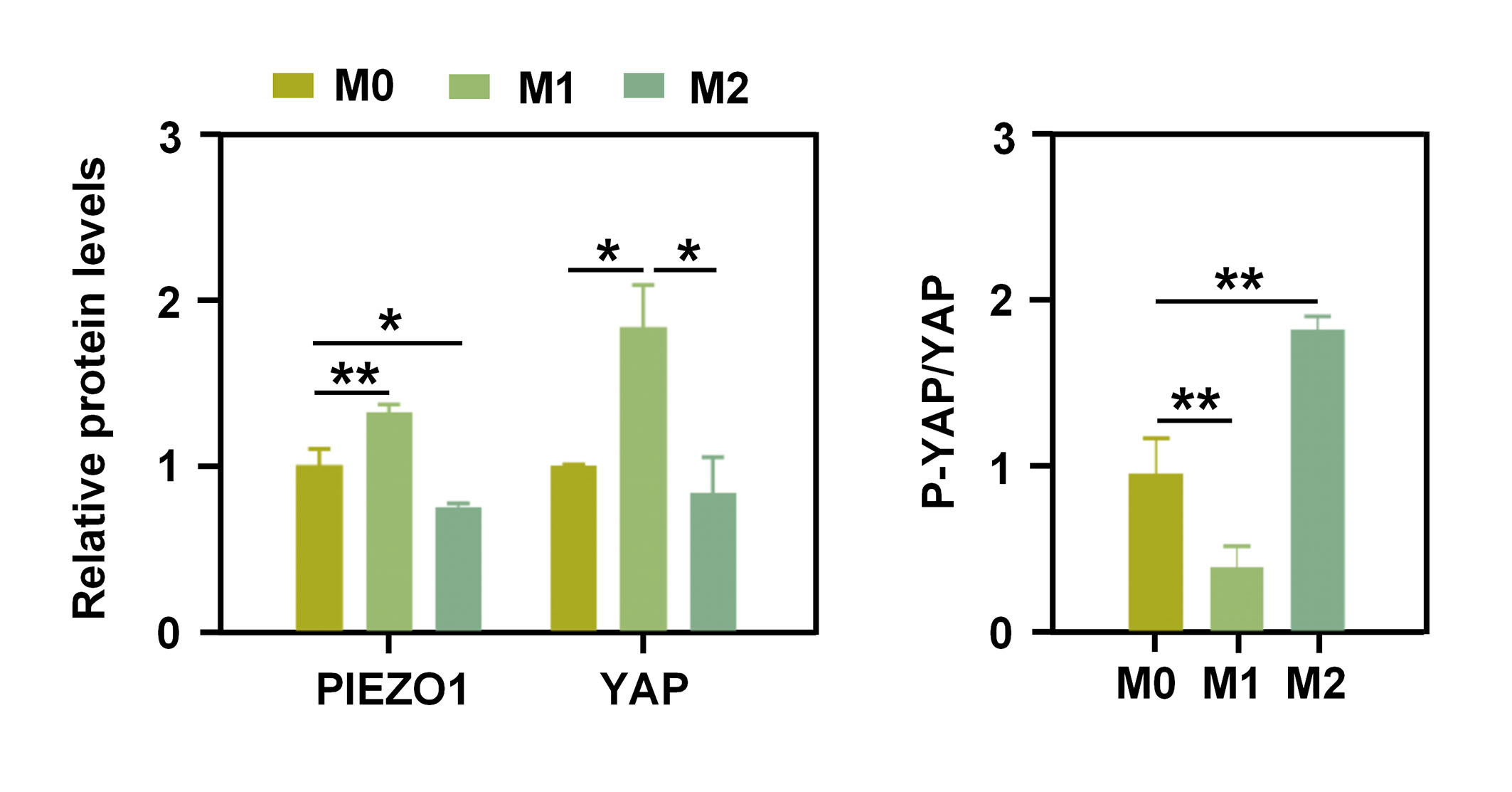
**

**Figure S3.** Quantitative analysis of protein expression levels in Figure 2C (*P<0.05, **P<0.01).


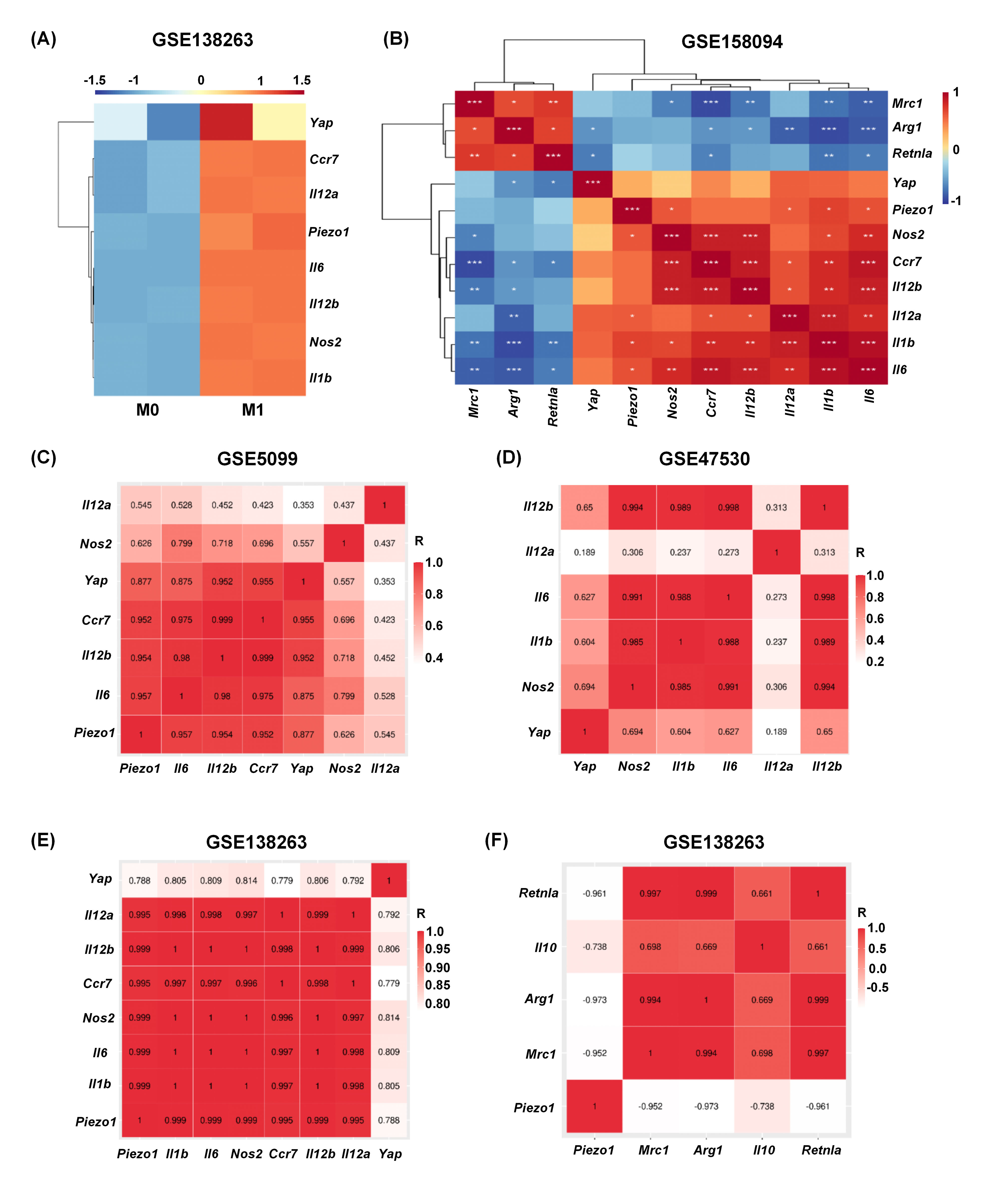


**Figure S4.** (A) Gene expression heatmap of Piezo1, Yap, and canonical marker genes of macrophage M1 phenotype in microarray dataset GSE138263. (B) Correlation heatmap of Piezo1, Yap, and canonical marker genes of macrophage M1/M2 phenotypes in RNA-seq dataset GSE158094. (C-E) Correlation heatmaps of Piezo1, Yap, and canonical marker genes of macrophage M1 phenotype in microarray datasets GSE5099, GSE47530, and GSE138263. (F) Correlation heatmap of Piezo1 and canonical marker genes of macrophage M2 phenotype in microarray dataset GSE138263.


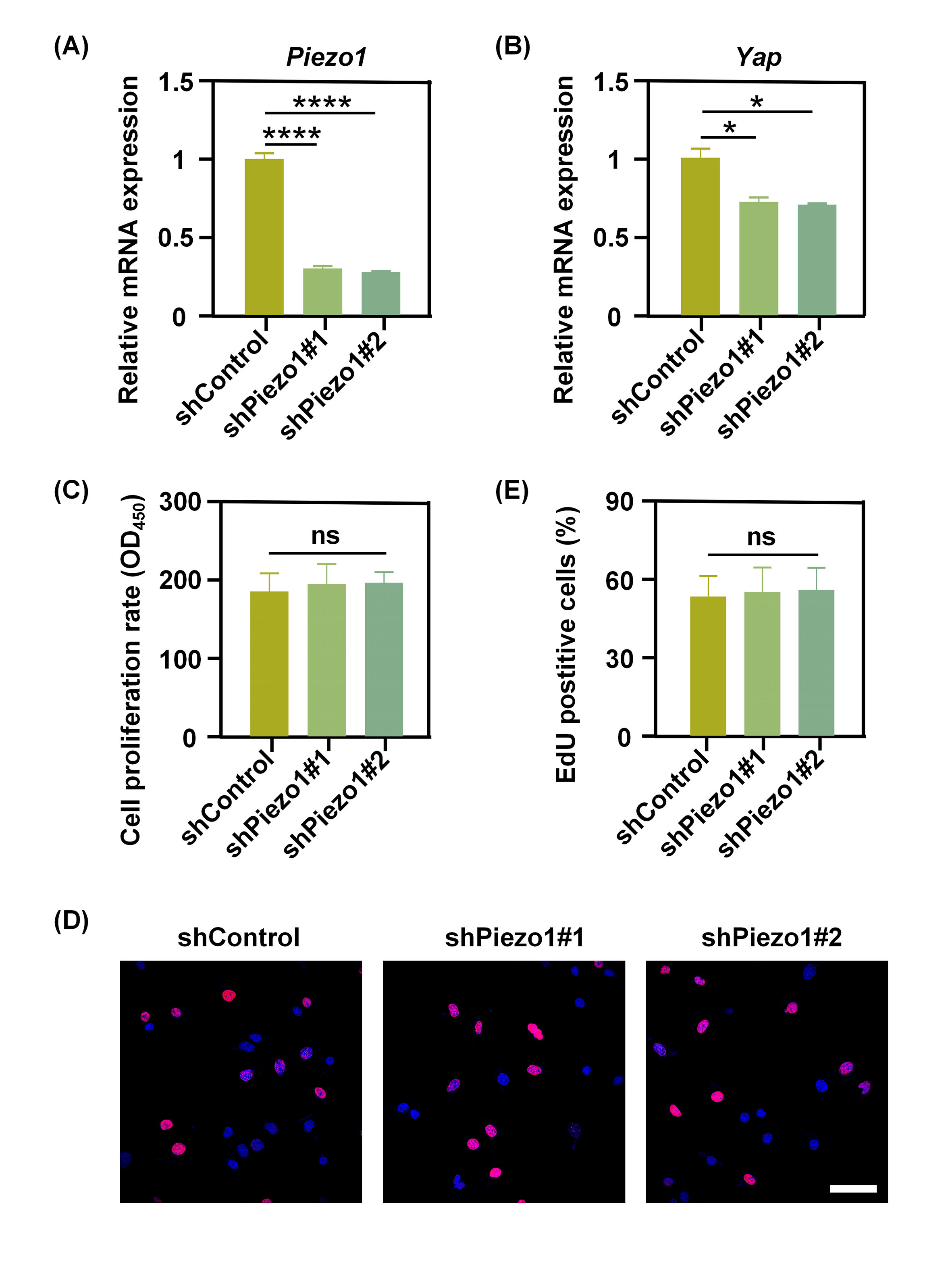


**Figure S5.** (A) Piezo1 knockdown was verified by RT-PCR (****P<0.0001). (B) Downregulation of YAP mRNA expression after knockdown of Piezo1 (*P<0.05). (C-E) The proliferation capacity of shControl and shPiezo1 cells was evaluated using CCK8 (C) and EdU assays (D-E) (Scale bar: 75 um).


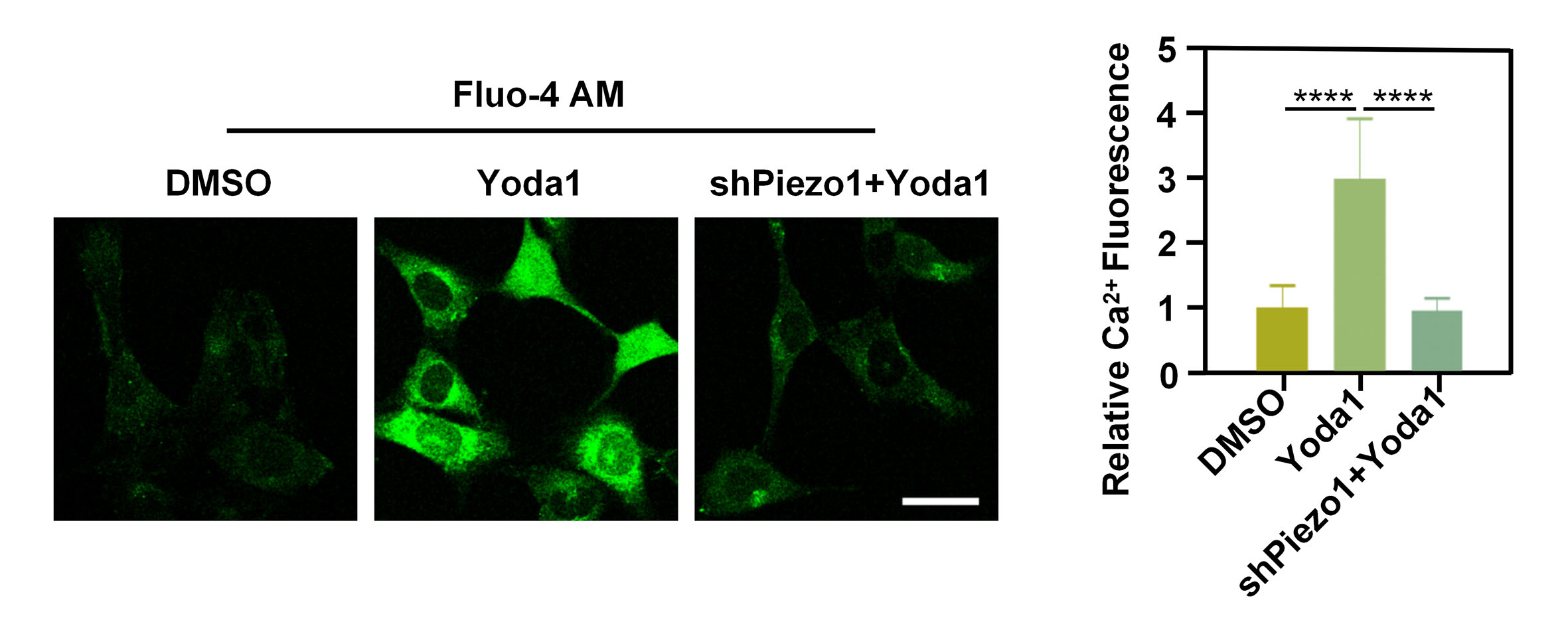


**Figure S6.** Representative fluorescence images of Fluo-4 AM-loaded BMDM and the quantitative fluorescence intensity of Fluo-4 AM (Scale bar: 30 um) (****P<0.0001).


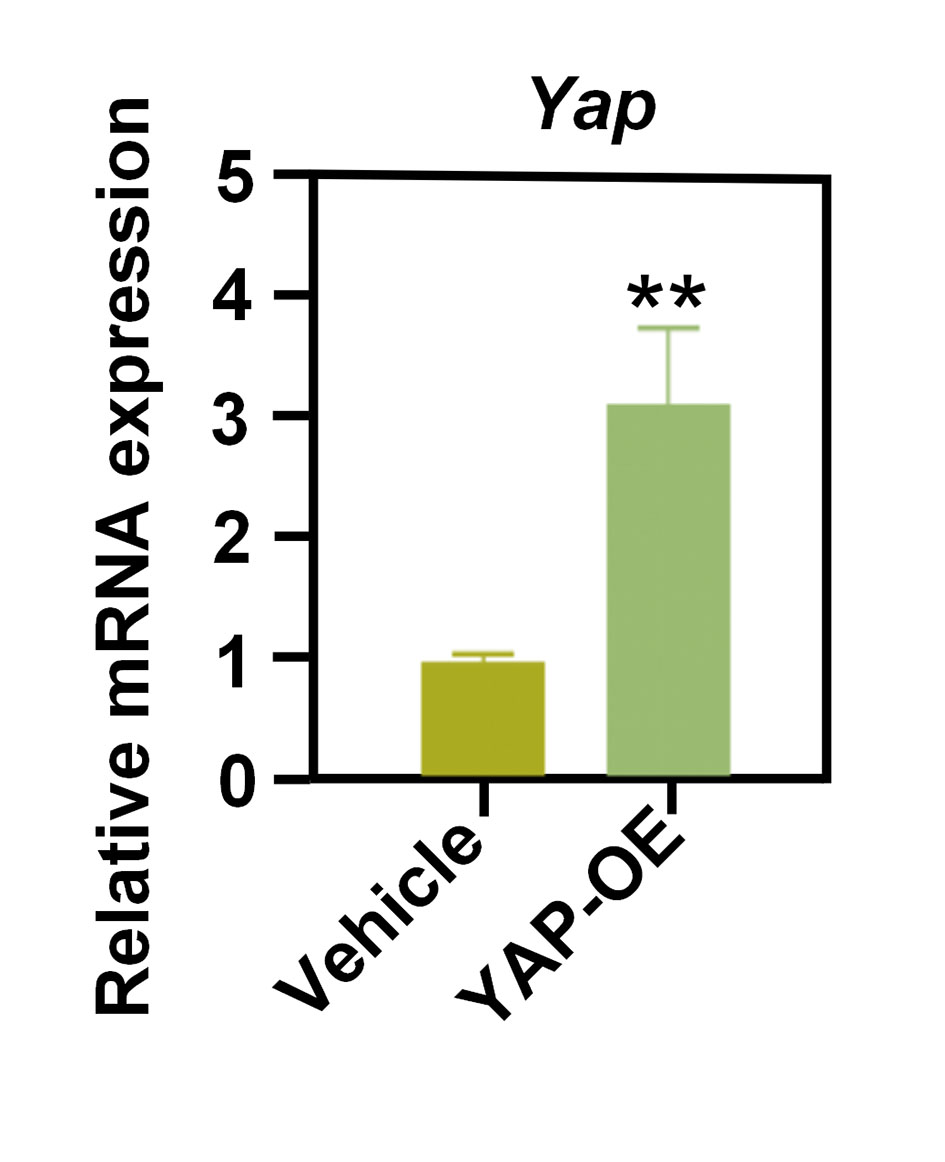


**Figure S7.** The transfection efficiency of the YAP overexpression plasmid was verified by RT-PCR (**P<0.01).


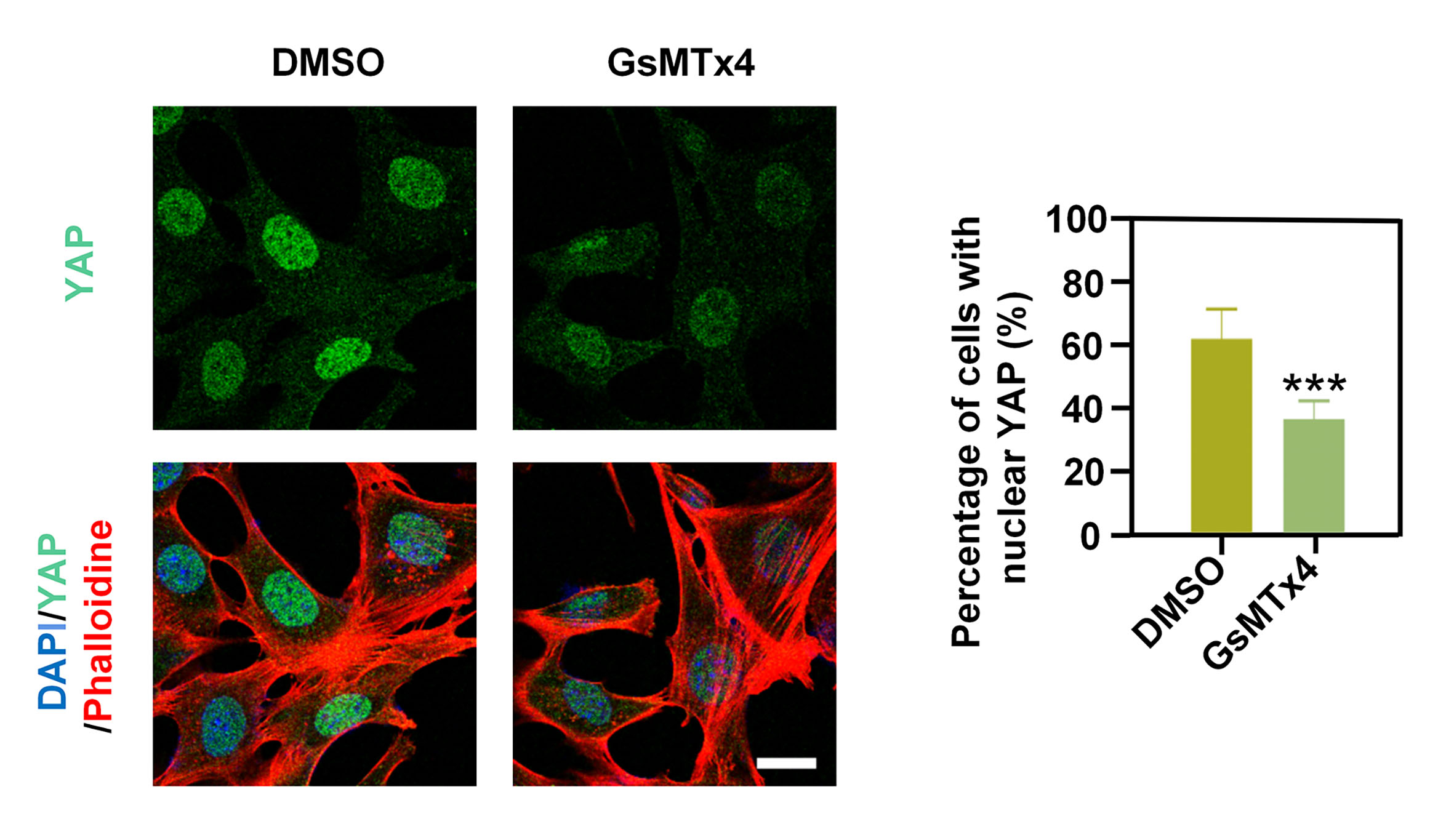


**Figure S8.** YAP immunofluorescence staining after GsMTx-4 treatment on the stiff PA gels (Scale bar: 20 um) (***P<0.001).


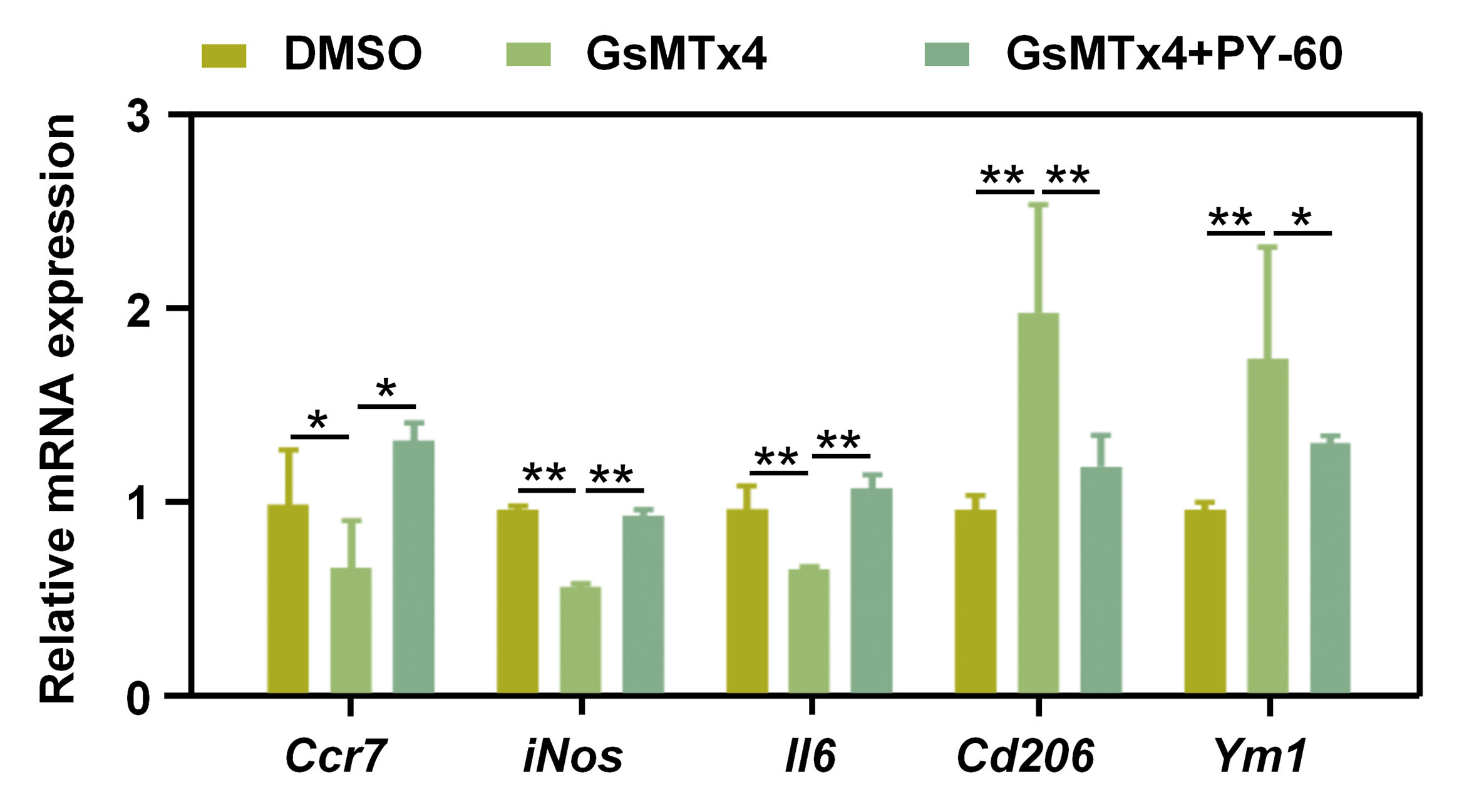


**Figure S9.** Relative mRNA expression levels of M1/M2-associated genes after DMSO, GsMTx4, GsMTx4+PY-60 treatment when cultured on stiff substrates (*P<0.05, **P<0.01).


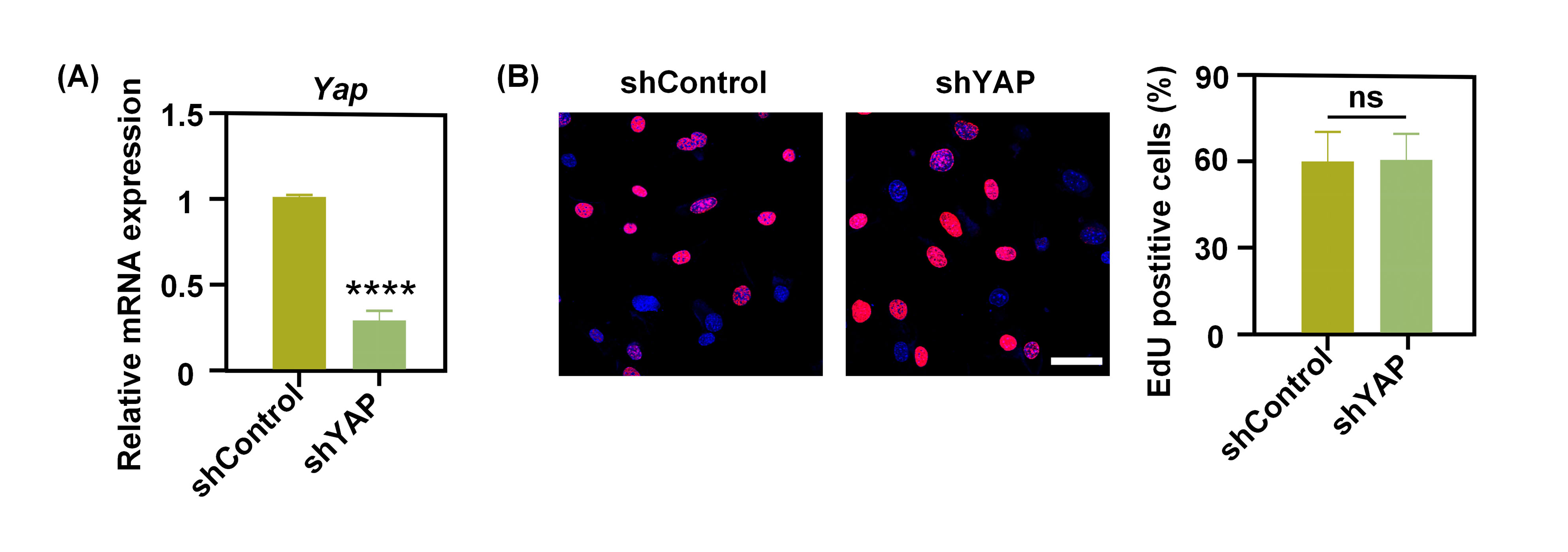


**Figure S10.** (A) Validation of YAP knockdown (****P<0.0001). (B) EdU assay was performed to evaluate the proliferative potentials of shControl and shYAP BMDM (Scale bar: 75 um).


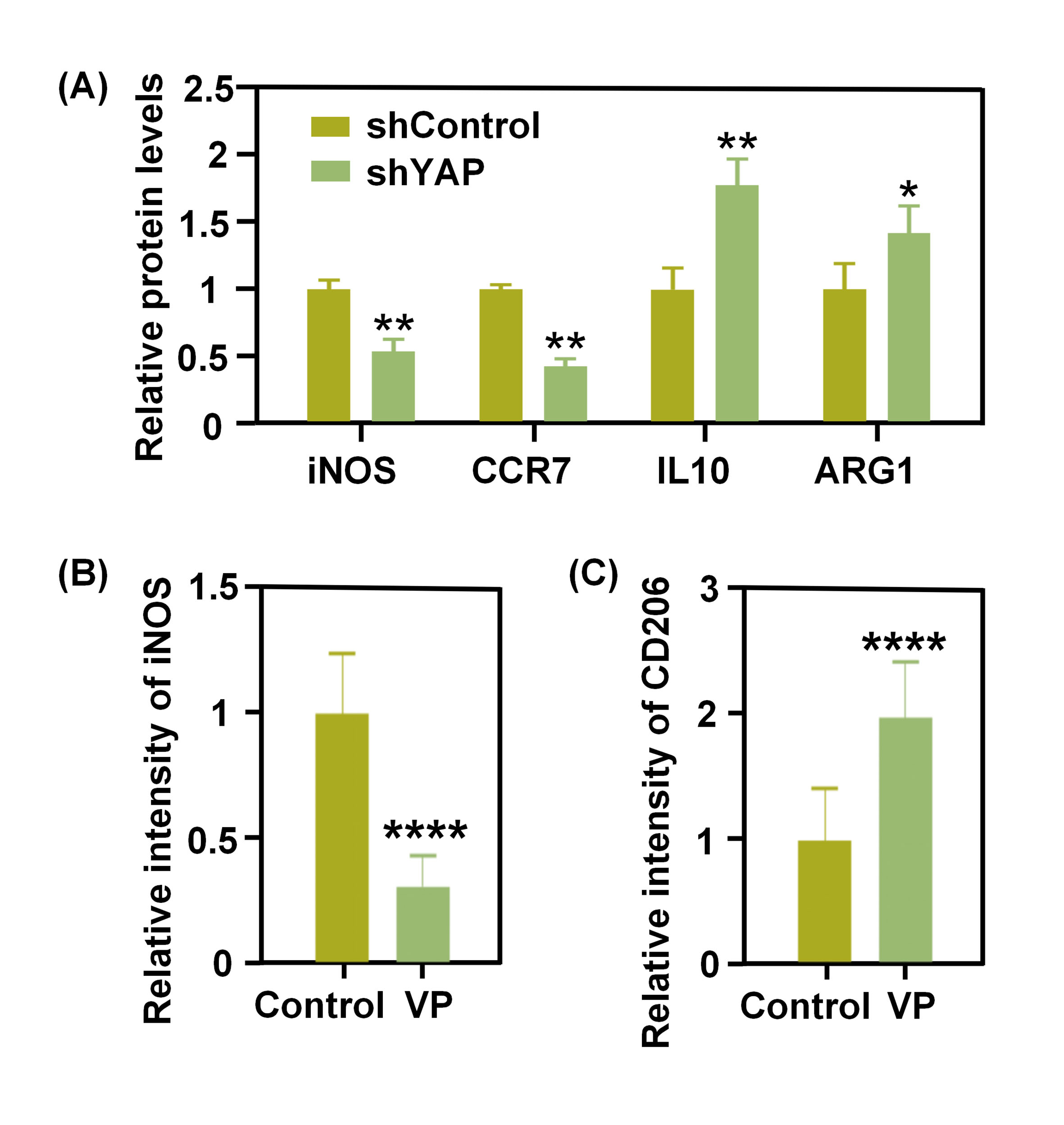


**Figure S11.** (A) Quantitative analysis of the protein expression in Figure 6B (*P<0.05, **P<0.01). (B-C) The statistical analysis of fluorescence intensities of iNOS (B) and CD206 (C) in Figure 6I (****P<0.0001).


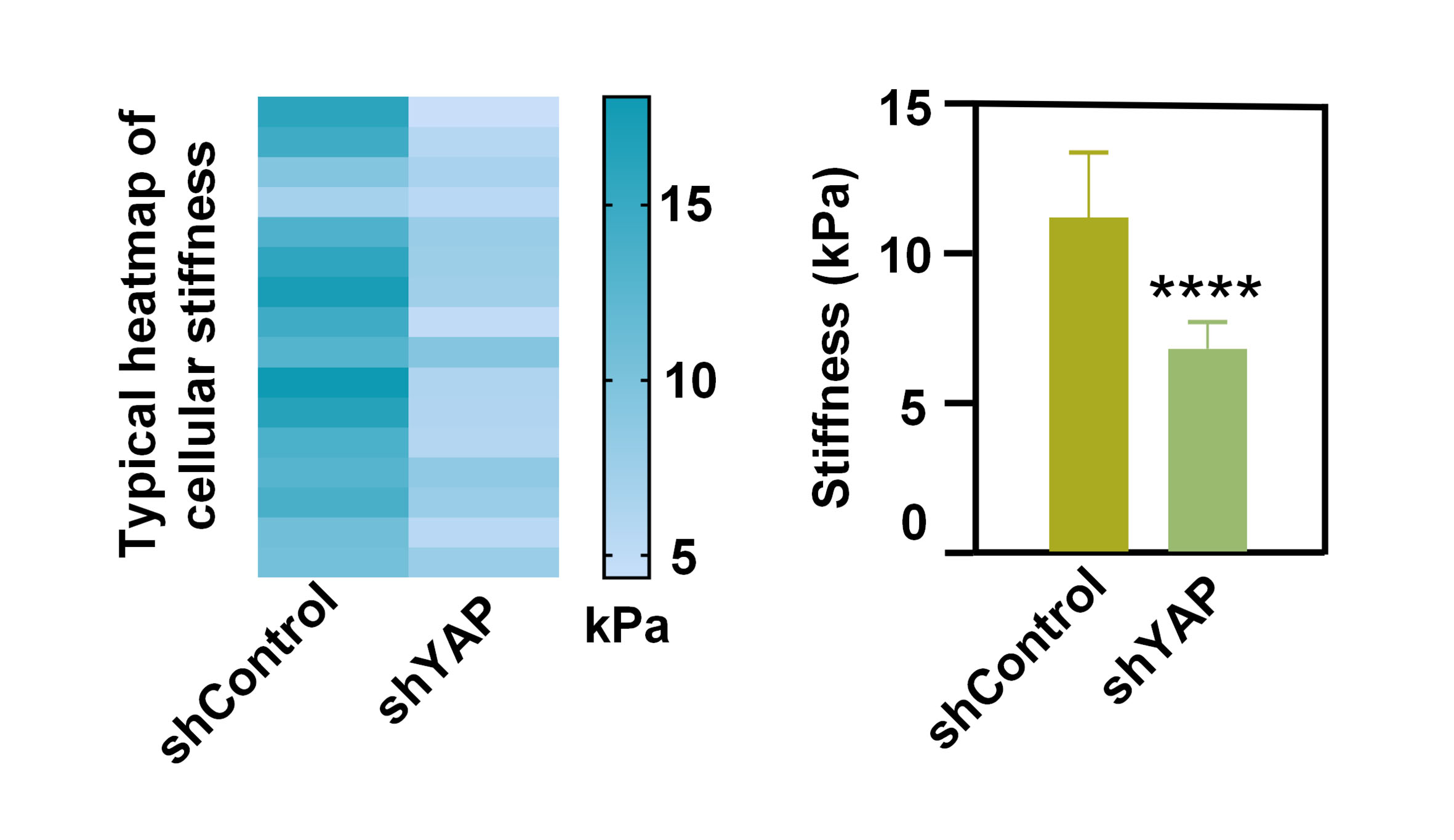


**Figure S12.** Quantitative statistics of stiffness of the shControl and shYAP BMDM cultured on stiff substrates, together with representative heatmaps (****P<0.0001).


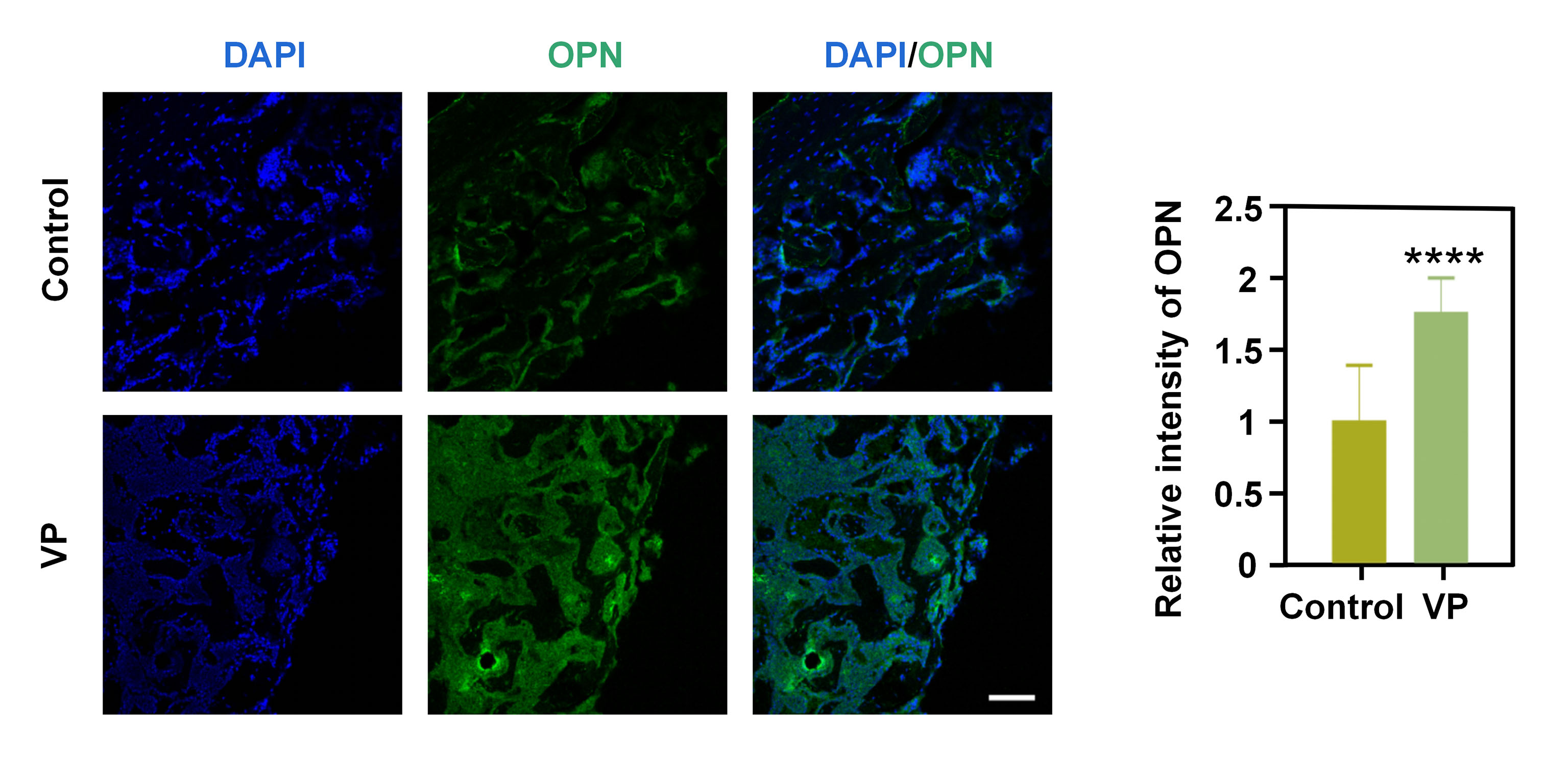


**Figure S13.** Representative images of immunofluorescence staining for osteopontin in femoral bone tissue and quantitative analysis of fluorescence intensity (Scale bar: 50 um) (****P<0.0001).

**Table S1. Primer sequences used for quantitative real-time PCR analysis.**

Target gene Forward sequence (5′-3′) Reward sequence (5′-3′)

*Gapdh* ATGGGTGTGAACCACGAGA CAGGGATGATGTTCTGGGCA

*Ym1* TGGAATTGGTGCCCCTACAA CCACGGCACCTCCTAAATTG

*Il12* CGAAACCTGCTGAAGACCAC AGCTCCCTCTTGTTGTGGAA

*Cd206* AACAAAGGGACGTTTCGGTG TCCTTCTGCCCAATGTTTGC

*Ccr7* TCATCCGTACCTTGCTCCAG ACAAGAAAGGGTTGACGCAG

*Tnf-α* CGTCAGCCGATTTGCTATCT CGGACTCCGCAAAGTCTAAG

*Il-1β* TCAGGCAGGCAGTATCACTC AGCTCATATGGGTCCGACAG

*iNos* TTGGCTCCAGCATGTACCCT TCCTGCCCACTGAGTTCGTC

*Arg1* ATCGTGTACATTGGCTTGCG CGTCGACATCAAAGCTCAGG

*Il10*  GCTGGACAACATACTGCTAACCG CACAGGGGAGAAATCGATGACA

*Piezo1* TCCACTAGCCATGCAGTTCA TCCAGGTAAAGCGCAGTTCA

*Yap* ACCCTCGTTTTGCCATGAAC TTCAACCGAGTCTCTCCTT
